# Physicochemical and Toxicological Characterization of Airborne Brake Wear Particles Reveals Oxidative Stress–Mediated DNA Damage

**DOI:** 10.1021/acs.est.5c10783

**Published:** 2026-02-03

**Authors:** Samuel Hyman, Siriel Saladin, Yurii Tsybrii, Oleksii Nosko, Matthew Williams, Alexander Zherebker, Kelvin Risby, David Topping, Adam Boies, Chiara Giorio, Martin Roursgaard, Peter Møller

**Affiliations:** † Department of Earth and Environmental Science, Centre for Atmospheric Science, School of Natural Sciences, 5292 The University of Manchester, Manchester M13 9PL, United Kingdom; ‡ Section of Environmental Health, Department of Public Health, University of Copenhagen, Copenhagen K 1014, Denmark; § Yusuf Hamied Department of Chemistry, 2152 University of Cambridge, Cambridge CB2 1EW, United Kingdom; ∥ Faculty of Mechanical Engineering and Ship Technology, 175570 Gdansk University of Technology, Gdansk 80-233, Poland; ⊥ Department of Engineering, 1980 University of Cambridge, Cambridge CB2 1PZ, United Kingdom

**Keywords:** nonexhaust emissions, Euro 7, PM_2.5_, PM_10_, particulate matter, chemical composition, toxicity, comet assay

## Abstract

Brake wear particles (BWP) are a significant source of urban air pollution, yet the toxicity linked to their chemical composition remains poorly understood. While studies have examined either chemical composition or toxicity, comprehensive investigations combining both remain limited. Here, we conducted an in-depth physicochemical characterization of airborne, size-separated BWP from two brake pad types and comprehensively assessed their *in vitro* toxicity using human lung epithelial cells (A549). Iron, primarily in the form of iron oxide, was the most abundant element in the wear particles (33–50% by mass), with evidence pointing to the brake disc as the main source. A surprisingly high resemblance in elemental composition at the nano- and microscale was observed. This, along with an absence of clear differences in metal profiles or toxicological responses between size fractions, suggests that brake wear microparticles may form through compaction of vapor-condensed nanoparticles on the friction surfaces, followed by their release through mechanical shearing. Acellular and cellular assays showed the concentration-dependent ability of all studied particles to induce reactive oxygen species production, antioxidant depletion, and oxidative stress-mediated DNA damage. The nonasbestos organic pad, with more than 50-fold higher copper levels than the low-metallic pad, induced stronger DNA damage and acellular antioxidant depletion, suggesting copper as a potential source for the enhanced toxicity.

## Graphical abstract

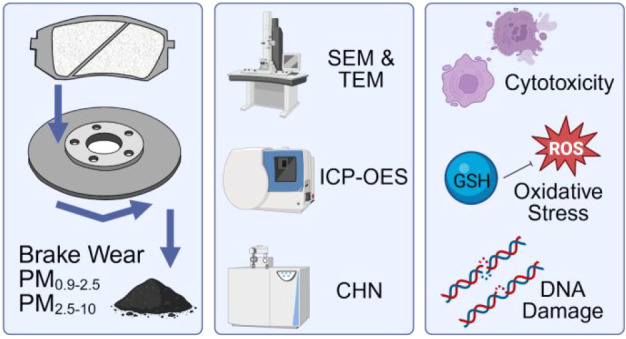

## 1 Introduction

The World Health Organization attributed 6.7 million deaths in 2019 to air pollution.^1^ Road traffic in 2021 is reported to cause 8% of the European Union’s emissions of airborne particles with aerodynamic diameters below 2.5 μm (PM_2.5_) as well as 10 μm (PM_10_).^2^ Both the European Environment Agency^3^ and the United States Environmental Protection Agency^4^ list automotive brakes as a relevant source of PM_2.5_ and PM_10_ from road traffic. In consequence, the European Union included brake wear PM_10_ limits of 3 and 7 mg/km/vehicle in the upcoming Euro 7 emission regulation.^5^ Brake wear particles (BWP) from disc brakes are emitted from the brake rotor and pads during braking^6–9^ as well as during acceleration and cruising,^10–12^ the latter potentially caused by drag torque.^13,14^ Although transport electrification is expected to reduce wear rates of disc brakes due to regenerative braking,^15,16^ the impact of electric vehicles on the emission rate, particle size distribution, chemical composition, and toxicity of airborne BWP remains unclear.

Brake rotors are often made from gray cast iron, while brake pads typically contain binders, reinforcing fibers, fillers, lubricants, and abrasives.^17^ Although there are no uniform definitions, brake pads are widely classified into low-metallic (LM), nonasbestos organic (NAO), and semimetallic (SM) types, among others.^18^ In practice, these classifications are based on manufacturer specifications and broad compositional characteristics rather than standardized criteria. The formulation of commercially available brake pads is usually proprietary. This hampers more detailed classification of brake pads, complicates comparisons between studies, and adds complexity to risk assessments.

Diameters of airborne BWP are reported to range from less^19–22^ than 10 nm to more^10,23–26^ than 5 μm, making them relevant to lung health. Particle deposition in the lungs is size-dependent, highlighting particle size as an important factor for health risk assessments. Coarse particles with aerodynamic diameter 2.5–10 μm can reach the lungs, whereas fine particles (i.e., <2.5 μm), and ultrafine particles (<100 nm) can penetrate deeper into the smaller airways.^27^ BWP are reported to contain heavy metals and organic compounds such as polycyclic aromatic hydrocarbons (PAHs) raising health concerns.^28,29^ Studies have shown that BWP have the potential to trigger a proinflammatory response in humans.^30–34^ Prolonged inflammation resulting from such responses may lead to chronic diseases, including cardiovascular and respiratory conditions, as well as cancer.^35^

The literature on the toxicological effects of BWP remains sparse, particularly regarding oxidative stress and genotoxicity in mammalian cell lines. Oxidative stress arises when stressors lead to overproduction of reactive oxygen species (ROS), altered oxidant defense system activity, and associated oxidation damage of biomolecules such as lipids, proteins or DNA.^36^ Direct comparison of the available studies is challenging due to the diverse methods employed, including differences in BWP generation techniques, brake-pad compositions, particle size fractions, exposure systems, concentrations, and toxicological assays. BWP that have been toxicologically investigated are often not subjected to extensive chemical characterization, and vice versa, highlighting the need to integrate toxicological and chemical analyses to facilitate interpretation across studies.

More research is needed to clarify the health risks of brake emissions, as concluded in a recent review by Christou et al.^37^ The research gap is addressed by our study using human alveolar epithelial cells (A549) and chemically characterized BWP from a commercially available gray cast iron disc and two types of automotive brake pads (LM and NAO). To our knowledge, this is the first study to collect airborne BWP in multiple size fractions and to combine comprehensive chemical and microscopic characterization with both cellular and acellular toxicological assessments relevant to human health. This includes the first application of the comet assay to evaluate DNA strand breaks caused by BWP, offering a more sensitive and mechanistic insight into the genotoxic effects.

## 2 Methods

Table S1.1 provides an overview of all performed experiments.

### 2.1 Particle Generation and Collection

Airborne wear particles were generated using a pin-on-disc tribometer with a brake pad pin on a gray cast iron disc, as outlined in Section S2. A sliding speed of 2 m/s and a contact pressure of 0.5 MPa resulted in typical steady-state pad temperatures below 100 °C (Figure S3.1A). These parameters approximate the average sliding speed of a passenger car uniformly decelerating from 36 km/h to zero, characteristic of urban driving where human exposure to BWP is most likely. For comparison, mean nominal contact pressures of 0.4 MPa have been reported for the Los Angeles City Traffic (LACT) cycle,^38^ and mean disc temperatures below 100 °C for the Worldwide Harmonized Light Vehicles Test Procedure (WLTP) cycle.^39^ Both LACT and WLTP are standardized laboratory procedures designed to reproduce real-world braking conditions.

The particles were collected with an electrical low pressure impactor from Dekati (ELPI+) equipped with ungreased aluminum substrates. The impactor stages were pooled into two size fractions, hereafter referred to as fine (0.94–2.5 μm) and coarse (2.5–10 μm). BWP collected on the lower impactor stages (16–940 nm) yielded insufficient mass for pooling and, consequently, for toxicological assessment. Therefore, the toxicological evaluation of brake wear nanoparticles is beyond the scope of the present study. Nevertheless, the collected amount of ultrafine particles was sufficient for electron microscopy, providing insights into their size, morphology, and elemental composition.

### 2.2 Physicochemical Characterization

The acid-digestible metal content was quantified using inductively coupled plasma optical emission spectroscopy (ICP-OES) upon digestion with hydrochloric acid and nitric acid. The mass fractions of combustible carbon, hydrogen, and nitrogen were determined by carbon, hydrogen and nitrogen (CHN) analysis. Oxygen, silicon, and sulfur were quantified using scanning electron microscopy (SEM) coupled with energy-dispersive X-ray spectroscopy (EDS). Transmission electron microscopy (TEM), scanning transmission electron microscopy (STEM), and EDS were used to assess particle shape and composition on a submicron level. More details for chemical characterization are provided in Section S4.

### 2.3 Cellular and Acellular Assays

Acellular antioxidant depletion was monitored using mass spectrometry analysis of aliquots collected from the incubation of BWP in a surrogate epithelial lung fluid^40^ containing 35 mg/L ascorbic acid (AA), 24 mg/L cysteine (CYS), and 62 mg/L glutathione (GSH) at physiological conditions, as outlined in Section S5. For all cellular assays, we used A549 cells cultured following standard protocols (Method S6.1). Cytotoxicity of BWP was assessed using the water-soluble tetrazolium salt 1 (WST-1) and lactate dehydrogenase (LDH) assays to ensure that the selected concentrations were not excessively toxic to cells over the 24 h exposure period (Method S6.2). Intracellular GSH and ROS levels were measured using a ThioGlo-1 (Method S6.3) and 2′,7′-dihydrofluorescein diacetate fluorescence assay (Method S6.4), respectively. DNA strand breaks were assessed using the standard alkaline comet assay (Method S6.5). In a separate experiment, the cell culture medium was supplemented with *N*-acetylcysteine (NAC) 10 mM at the start of the 24 h exposure period. Oxidatively damaged DNA was assessed in a separate experiment using the Fpg-linked comet assay. The results have been analyzed using ANOVA tests on separate samples (e.g., NAO fine) and adjusted for interexperiment variation (with Sidak posthoc test for differences between exposure groups). The results are reported as mean and standard error of the mean (SE). Net effects of biomarkers and 95% confidence interval (95% CI) are reported to give an impression of the effect size and experimental variation. Comparisons of differences in effects between particles have been assessed by nonoverlapping confidence intervals of effect sizes. We only report differences when there are nonoverlapping CIs between samples. Statistical analyses were performed using GraphPad Prism version 10.0.0 for Apple Mac, GraphPad Software, Boston, Massachusetts USA.

## 3 Results

### 3.1 Particle Generation and Collection

For BWP generated with both the low-metallic (BWP_LM_) and nonasbestos organic (BWP_NAO_) pads, approximately 300 mg of PM_10_ was collected in each case (Table S7.1). The fine (0.94–2.5 μm) and coarse (2.5–10 μm) BWP masses were sufficient for extensive chemical analysis and toxicological assays. The steady-state coefficients of friction (COF) were approximately 0.5 for the LM and 0.35 for the NAO brake pad (Figure S3.1B), which were used to calculate dissipated frictional energies.

### 3.2 Physicochemical Characterization

#### 3.2.1 Particle Size

Real-time measurements by number using the ELPI+ indicated modes with aerodynamic diameters of approximately 0.3 μm and 4 μm for both BWP_LM_ and BWP_NAO_. In both cases, stage 13 (3.6–5.3 μm) yielded the highest collected masses (Table S7.1). Corresponding microscopic images for different size fractions are provided in Figures S8.1 and S8.2 along with online size distribution data in Figure S3.1C.

Particles with Feret diameters up to 10 μm as well as submicron particles were found in the coarse fractions (Figure 1). The Feret diameter refers to the maximum distance between two parallel lines tangent to opposite sides of a particle. Experiments with TEM imaging showed nanoparticles with spherelike shapes in the order of 10 nm found in BWP_LM_ (Figure 1C) and BWP_NAO_ (Figure S8.3C).

**Figure 1:**
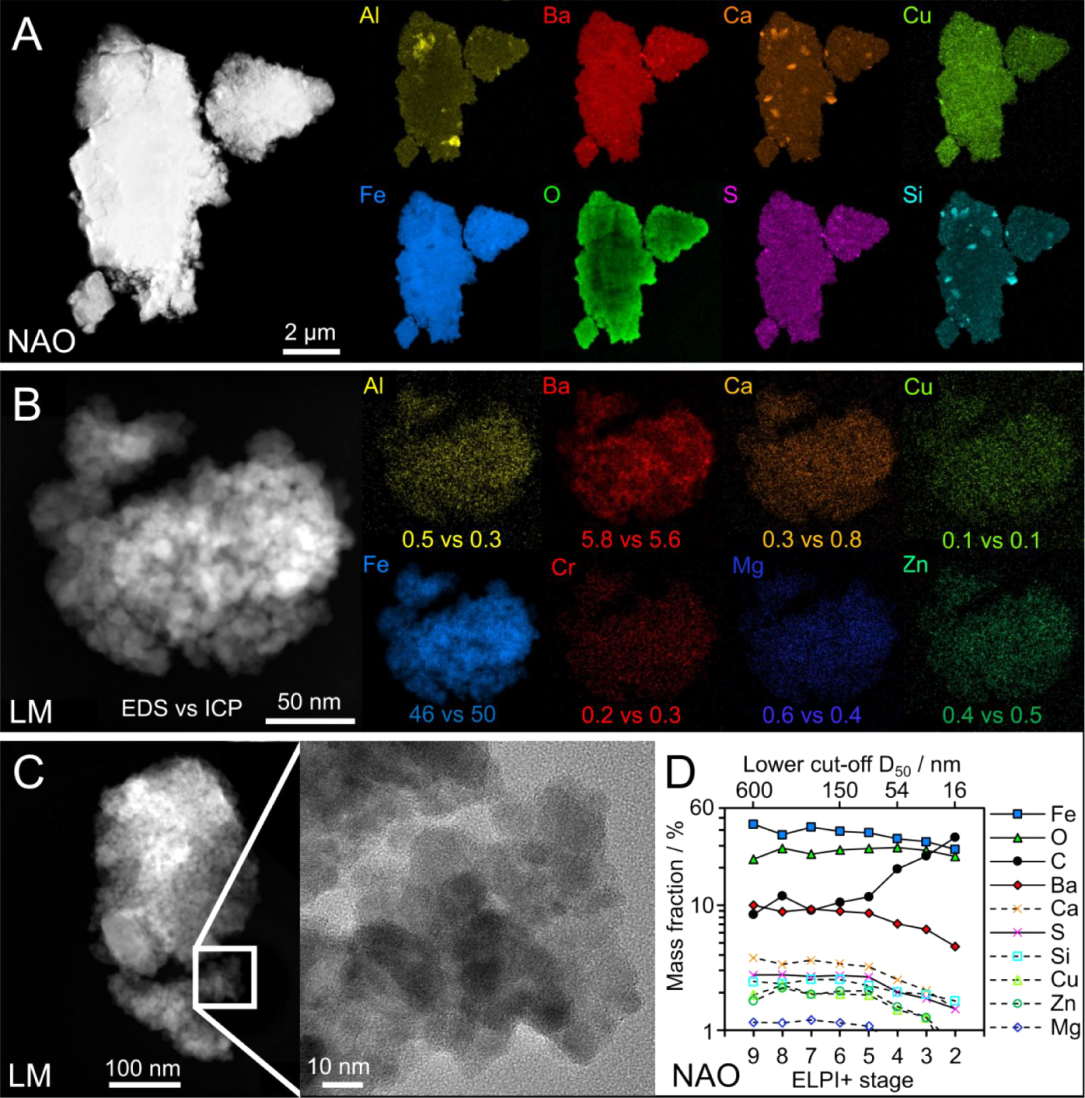
High-angle annular dark-field STEM images and EDS maps of particles from the coarse fraction of (A) BWP_NAO_ and (B, C) BWP_LM_. Panel (B) includes the elemental mass fractions (in %) according to STEM-EDS for the shown particle cluster versus ICP-OES for the bulk. (D) Particle size-resolved elemental composition of BWP_NAO_ measured by SEM-EDS, excluding Al due to the aluminum substrates.

Dynamic light scattering (DLS) of aqueous BWP dispersions revealed polydispersity indices (PDI) of 0.70 ± 0.17 and 0.68 ± 0.05 for the fine and coarse BWP_LM_ as well as 0.54 ± 0.05 and 0.65 ± 0.05 for BWP_NAO_, respectively. The standard deviations refer to triplicates. Hydrodynamic particle diameters are not provided due to the high PDI, which indicates a broad size distribution and limits the reliability of DLS-derived size values.

#### 3.2.2 Chemical Composition

Characterization with ICP-OES and SEM-EDS showed iron as the most abundant element by mass in all investigated cases of BWP (Table 1). The elements Ba, C, and O were detected in BWP with mass fractions exceeding 5%. Other elements found with mass fractions greater than 1% were Al, Ca, Cu, Mg, S, Si, and Zn, depending on the brake pad. A total of 90% of the fine and coarse BWP mass is explained by the detected elements. This estimate, however, should be taken with caution given that the measurement for oxygen is based on SEM-EDS which is a semiquantitative surface technique.

**Table 1:** Measured Elemental Mass Fractions According to CHN, ICP-OES, and SEM-EDS Analysis in Percent for the Brake Disc, the Low-Metallic (LM), and Non-Asbestos Organic (NAO) Brake Pad as Well as Fine (0.94–2.5 μm) and Coarse (2.5–10 μm) BWP^a^

	Brake disc	Brake pad		BWP			
		LM	NAO	LM		NAO	
				Fine	Coarse	Fine	Coarse
C^b^	3.1 ± 0.1	24.5 ± 0.1	20.5 ± 0.1	7.0 ± 0.1	6.7 ± 0.1	7.0 ± 0.1	7.1 ± 0.1
H^b^	<0.1	0.6 ± 0.1	1.1 ± 0.1	0.5 ± 0.1	0.5 ± 0.1	0.7 ± 0.1	0.7 ± 0.1
N^b^	<0.1	0.4 ± 0.1	0.3 ± 0.1	0.1 ± 0.1	0.1 ± 0.1	0.1 ± 0.1	0.1 ± 0.1
O^c^	3.6 ± 0.3	18 ± 1	20 ± 1	22 ± 1	22 ± 1	26 ± 1	24 ± 1
S^c^	0.1 ± 0.0	3.9 ± 0.1	4.8 ± 0.2	1.5 ± 0.2	1.4 ± 0.1	2.5 ± 0.3	2.6 ± 0.2
Si^c^	1.2 ± 0.1	2.2 ± 0.6	2.4 ± 0.4	1.3 ± 0.1	1.2 ± 0.1	2.2 ± 0.2	2.5 ± 0.3
Al^d^	<0.17	0.4 ± 0.1	6.9 ± 0.1	0.3 ± 0.1	0.3 ± 0.1	3.7 ± 0.2	3.8 ± 0.1
Ba^d^	<0.11	8.5 ± 1.0	8.7 ± 0.6	5.3 ± 0.2	5.6 ± 0.4	7.9 ± 0.3	7.9 ± 0.1
Ca^d^	0.6 ± 0.1	1.4 ± 0.1	6.1 ± 0.1	0.8 ± 0.1	0.8 ± 0.1	3.9 ± 0.3	4.0 ± 0.1
Cr^d^	0.16 ± 0.01	<0.06	<0.06	0.27 ± 0.01	0.27 ± 0.02	0.05 ± 0.01	0.06 ± 0.01
Cu^d^	0.14 ± 0.01	<0.08	3.8 ± 0.1	0.08 ± 0.01	0.08 ± 0.01	1.8 ± 0.1	1.7 ± 0.1
Fe^d^	97 ± 3	22 ± 1	1.1 ± 0.1	50 ± 1	50 ± 3	32 ± 2	33 ± 1
K^d^	<0.03	0.20 ± 0.01	0.08 ± 0.01	0.09 ± 0.01	0.10 ± 0.01	0.06 ± 0.02	0.07 ± 0.01
Mg^d^	<0.06	1.0 ± 0.1	2.4 ± 0.1	0.4 ± 0.1	0.4 ± 0.1	1.2 ± 0.1	1.2 ± 0.1
Mn^d^	0.4 ± 0.1	0.06 ± 0.01	<0.05	0.21 ± 0.01	0.21 ± 0.02	0.14 ± 0.01	0.14 ± 0.01
Zn^d^	<0.06	0.9 ± 0.1	3.3 ± 0.1	0.4 ± 0.1	0.5 ± 0.1	2.0 ± 0.1	1.9 ± 0.1
Sum	106	84	82	90	90	91	91

a Uncertainty estimates refer to standard deviations. Detection limits are indicated with a less-than sign (<).

b CHN analyzer.

c SEM-EDS.

d ICP-OES.

The fine and coarse BWP showed similar elemental composition. All elements detected in the brake disc and brake pads were also detected in the airborne BWP. Twelve elements were not detected by ICP-OES in any sample. Their respective limits of detection (in parentheses) were: B (0.49%), Cd (0.06%), Co (0.06%), Li (0.21%), Mo (0.05%), Na (0.15%), Ni (0.07%), P (0.12%), Pb (0.10%), Sb (0.20%), Ti (0.05%), and V (0.53%). The detection limits, expressed as mass fractions, were calculated as the mean blank signal plus three times its standard deviation, normalized to the sample concentration after digestion and dilution.

Elemental mapping of individual BWP (Figure 1A) typically showed the coexistence of elements associated with the brake pad and the disc. Surprisingly, this was also the case when looking at clusters of nanoparticles. For example, the comparison between STEM-EDS versus ICP-OES showed similar mass fractions for the detected elements in case of coarse BWP_LM_ (Figure 1B): Al (0.5% vs 0.3%), Ba (5.8% vs 5.6%), Ca (0.3% vs 0.8%), Cr (0.2% vs 0.3%), Cu (0.1% vs 0.1%), K (0.1% vs 0.1%), Fe (46.0% vs 50.1%), Mg (0.6% vs 0.4%), and Zn (0.4% vs 0.6%). Figure S8.3 shows an example for coarse BWP_NAO_: Al (3.9% vs 3.8%), Ba (9.7% vs 7.9%), Ca (4.3% vs 4.0%), Cu (1.4% vs 1.7%), Fe (47.2% vs 33.1%), Mg (0.9% vs 1.2%), and Zn (2.1% vs 1.9%). Note that carbon is included in the mass balance for ICP-OES but not for STEM-EDS.

The particles showed a high degree of intra- and interparticle elemental homogeneity (Figure S8.4). SEM-EDS analysis of stage-separated BWP revealed a relatively consistent elemental composition across different particle sizes (Figures 1D and S8.5). The elemental ratios did not clearly change with particle size, except for carbon which was found to increase with decreasing particle size. For stage 2 (16–30 nm), the mass-dominating elements carbon, iron, and oxygen were found to spatially correlate with each other, implying submicron homogeneity (Figure S8.6).

The chemical nature of the nanoparticles was additionally examined by high-resolution TEM, which revealed lattice fringes with the most frequent spacings around 0.25 nm (Figure S8.7). The prevalence of spherelike, iron-rich nanoparticles prompted the study of elemental iron in a tube furnace, where nanoparticle formation was observed above approximately 1100 °C (Figure S9.1), without the involvement of mechanical abrasion or friction.

The surface of the worn NAO brake pad showed elevated iron abundance of 15% by mass compared to the 1% before wear according to SEM-EDS (Figure S8.8). Similarly, the brake disc surface after wear showed elements attributable to the NAO pad (Figure S8.9). CHN analysis indicated a carbon mass fraction of 3.1% in the brake disc, unevenly distributed within the iron matrix depending on the disc’s condition (Figure S8.10).

The iron from the BWP appears to be oxidized (Figure 1) in contrast to the iron from the original material. The LM pad contains fibers dominant in elemental iron with diameters of approximately 100 μm (Figure S8.11A). Copper was not found in the LM but in the NAO pad, where it was present as elemental copper fibers with diameters of approximately 100 μm (Figure S8.11B). Copper in BWP_LM_ and BWP_NAO_ seemed to be present as submicron particles with an unclear oxidation state. The BWP_NAO_ additionally showed copper-rich regions in particles with Feret diameters up to approximately 2 μm, where copper appeared to negatively correlate with oxygen (Figure S8.12).

### 3.3 Cellular and Acellular Assays

#### 3.3.1 Acellular Antioxidant Depletion

Both BWP_LM_ and BWP_NAO_ caused depletion of acellular AA, CYS, and GSH as well as formation of GSSG in surrogate epithelial lung fluid (Figure 2A for GSH; see Figure S10.1 for time-resolved data of all analytes). Notably, BWP_NAO_ caused significantly more depletion than BWP_LM_ with CYS and GSH being completely depleted already at the lowest tested concentration of 4 μg/mL. No clear difference was found between the fine and coarse size fractions.

**Figure 2:**
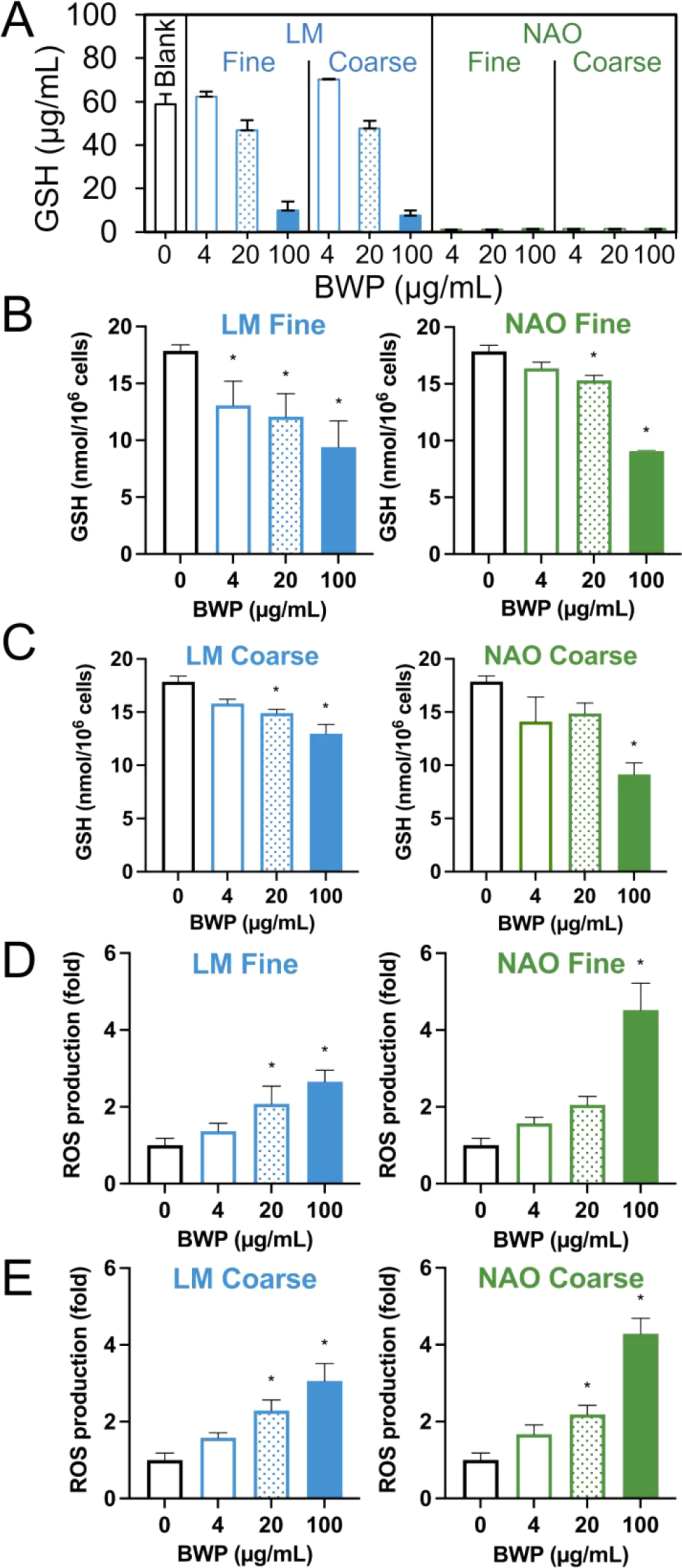
(A) Acellular levels of glutathione (GSH) in surrogate epithelial lung fluid after 3 h exposure to brake wear particles (BWP). Cellular levels of GSH after 24 h exposure to (B) fine and (C) coarse BWP. Intracellular reactive oxygen species (ROS) production after 3 h exposure to (D) fine and (E) coarse BWP. For cellular assays, bars and error bars are mean and SE of at least three independent experiments. *P < 0.05 compared to the negative control group. Positive controls significantly decreased GSH levels, or increased ROS production, respectively.

#### 3.3.2 Cytotoxicity

BWP_LM_ coarse and BWP_NAO_ fine and coarse caused cytotoxicity at the highest concentrations; however, cytotoxicity remained less than 35% of the negative control (Figures S11.1 and S11.2).

#### 3.3.3 Intracellular Glutathione Levels

Figure 2B–C shows the effect of BWP on intracellular GSH levels after 24 h exposure. All BWP samples showed a concentration–response effect which was statistically significant as assessed by ANOVA at least at the highest concentration. The positive control of 0.75 mM diethyl maleate (DEM) and 50 μM buthionine sulfoximine (BSO) reduced GSH with a mean difference of −8.43 (95% CI: −5.50, −11.36) nmol/10^6^ cells.

#### 3.3.4 Intracellular ROS Production

Figure 2D–E shows the effect of 3 h exposure on intracellular ROS production. All the BWP samples caused a concentration–response increase with significant results from either 20 μg/mL or 100 μg/mL, with the greatest response observed with BWP_NAO_. The positive control (100, 250, and 500 μM H_2_O_2_) significantly increased the intracellular ROS production with mean fold-changes of 1.84 (95% CI: 0.38, 3.30), 2.31 (95% CI: 0.85, 3.77), and 2.75 (95% CI: 1.56, 3.94), respectively.

#### 3.3.5 DNA Strand Breaks

Levels of DNA strand breaks were assessed by the comet assay after 24 h exposure (Figure 3A–B). Genotoxic effect of exposure to BWP showed a concentration–response effect in all cases which was statistically significant using ANOVA in at least one concentration per sample. Genotoxic effects were significantly higher in fine BWP_NAO_ samples (mean difference: 1.22, CI: 0.76, 1.68) compared to fine BWP_LM_ (mean difference: 0.31, CI: −0.05, 0.67) at 100 μg/mL (Figure 3A). The positive control (10, 50, and 100 μM H_2_O_2_) significantly increased DNA strand breaks with a mean difference of 0.67 (95% CI: 0.33, 1.02), 1.81 (95% CI: 1.31, 2.30), and 1.95 (95% CI: 1.52, 2.39) lesions/10^6^ base pairs, respectively.

**Figure 3:**
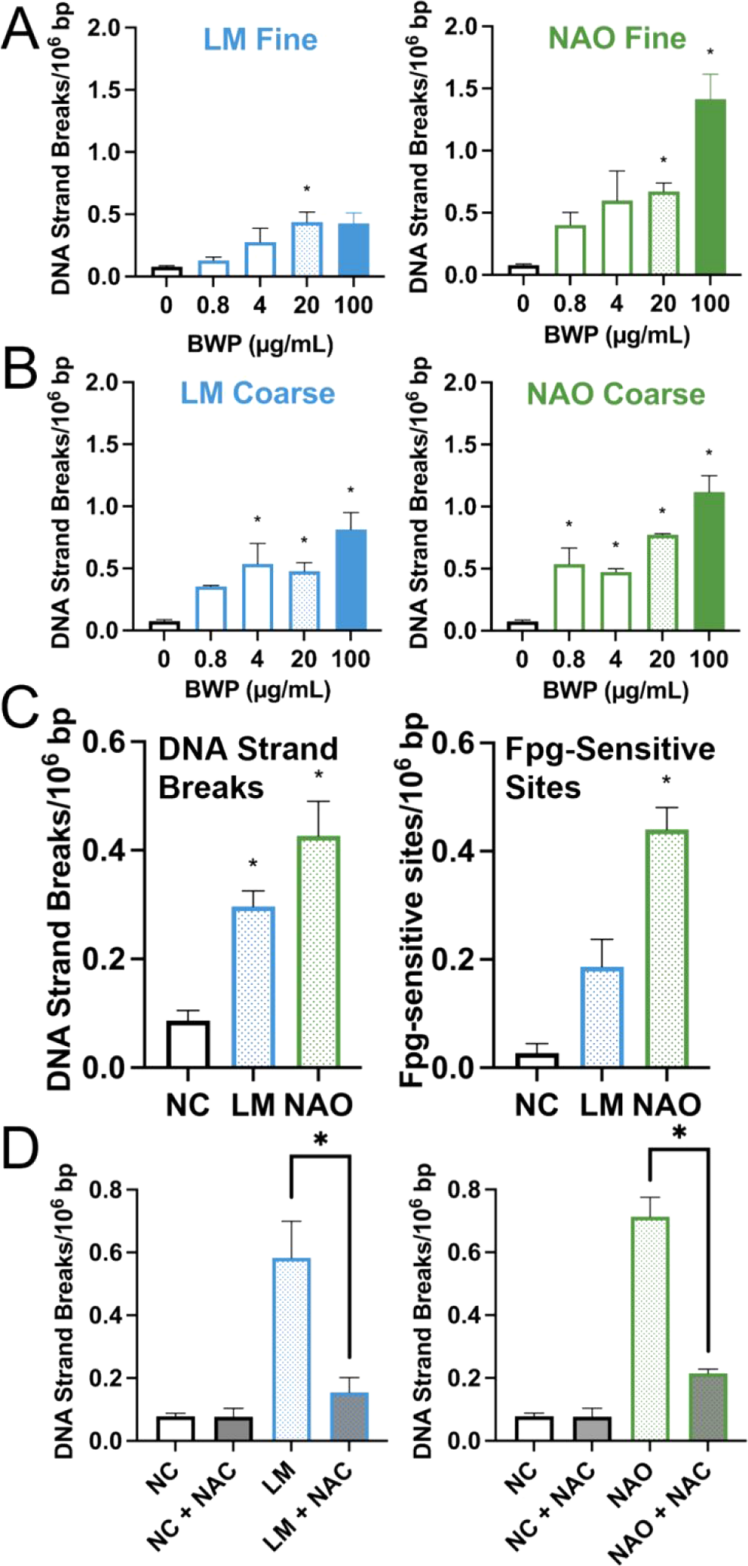
DNA strand breaks measured by the standard comet assay after 24 h exposure to (A) fine and (B) coarse brake wear particles (BWP). (C) Fpg-linked comet assay results after 24 h exposure to fine BWP at 20 μg/mL. (D) Effect of 10 mM *N*-acetylcysteine (NAC) supplementation on DNA strand breaks induced by 20 μg/mL fine BWP. All assays were performed with A549 cells. Bars and error bars are mean and SE of at least three independent experiments. *P < 0.05 compared to the negative control group for (A), (B), and (C) whereas (D) *P < 0.05 for interaction between fine BWP_NAO_ and NAC (i.e., NAC reduces the genotoxic effect of fine BWP_NAO_). NC: negative control.

#### 3.3.6 Oxidatively Damaged DNA

The Fpg-modified comet assay showed that fine BWP_NAO_ at 20 μg/mL could also induce another type of DNA damage, especially 8-oxoguanine nucleobase, which may give rise to mutations (Figure 3C). The hydrogen peroxide control (10 μM H_2_O_2_) increased Fpg-sensitive sites, with a mean difference of 0.27 (95% CI: −0.13, 0.67; *n* = 2) lesions/10^6^ base pairs. The assay control for Fpg treatment (4.5 mM potassium bromate) significantly increased Fpg-sensitive sites, with a mean difference of 0.83 (95% CI: 0.44, 1.22; *n* = 3) lesions/10^6^ base pairs.

#### 3.3.7 Effect of NAC Supplementation on DNA Strand Breaks

To assess the relationship between BWP exposure and DNA strand breaks, we supplemented A549 cell cultures with 10 mM NAC at the start of the exposure period. NAC supplementation significantly reduced DNA damage in cells exposed to 20 μg/mL of fine BWP_LM_ or BWP_NAO_ (*P* < 0.05 for interaction between fine BWP_LM_ or BWP_NAO_ and NAC exposure on levels of DNA strand breaks, Figure 3D).

## 4 Discussion

### 4.1 Particle Generation and Collection

Real-world driving involves frequent braking under varying loads, speeds, and temperatures. In this study, a single condition was used to generate sufficient particle mass for toxicological assays, reflecting average rather than the full range of real-world braking. The applied contact pressure (0.5 MPa) and pad temperature (80–90 °C) fall within reported ranges for light-duty braking,^38,39,41^ supporting the realism of our setup. However, the representativeness of pin-on-disc tribometers remains under discussion, and biological results should be considered indicative rather than exhaustive.

Alves et al.^29^ showed that different braking cycles with disc temperatures of 100–550 °C produced PM_10_ carbon mass fractions between 6.7% and 75% using the same NAO pads. Variations in temperature, collection method, contamination, particle size, and friction material or surface state (e.g., corrosion^42^) may affect particle composition and toxicity, highlighting the need for transparent generation protocols.

The environmental risk of BWP depends on both toxicity and exposure. The LM pad produced about twice as much particulate mass per unit time as the NAO pad. However, when normalized by the dissipated frictional energy, the two pads yielded more similar averages (0.042 and 0.033 mg/kJ), indicating comparable real-world emission potentials under these conditions.

### 4.2 Physicochemical Characterization

#### 4.2.1 Particle Size

Online particle number distributions (Figure S3.1C) showed two modes at aerodynamic diameters of approximately 0.3 and 4 μm for both brake pads. This pattern agrees with Kukutschová et al.,^43^ who reported similar results using a comparable pin-on-disc setup, where the ultrafine mode increased sharply above disc temperatures of approximately 250 °C. Such temperatures exceed those reported for the WLTP brake cycle, where final and peak disc temperatures remained below 100 and 200 °C, respectively.^39^ Accordingly, our experiments were conducted at lower temperatures.

The collected fine and coarse BWP cover a wide range of particle sizes as observed with DLS and microscopy. The extended collection period with ungreased substrates may have facilitated particle bounce and blow-off in the ELPI+.^44^ As a result, particles that would deposit on higher stages may instead be collected on lower stages. Figures S8.1 and S8.2 show particles in the fine fractions of LM and NAO with Feret diameters above 2.5 μm indicating that these particles may have bounced or blown-off from a previous stage. Similarly, the lowest stage (stage 2) showed particles with Feret diameters up to 500 nm indicating the occurrence of bouncing (Figure S8.6).

Bouncing or blow-off was shown for tire and road wear particles collected on lower stages even when using greased substrates,^45^ demonstrating the limitations of size-resolved particle collection with impactors over extensive time periods. Despite these uncertainties, the SEM analysis consistently showed that particle size decreased with decreasing stage number (Figures S8.1 and S8.2). The inorganic analysis showed no clear chemical difference between the fine and coarse BWP, which was also reported by Neukirchen et al.^46^ who collected brake wear PM_2.5_ and PM_10_ with cyclones.

#### 4.2.2 Chemical Composition

Clear differences in chemical composition between the LM and NAO brake pad were observed as shown in Table 1. For example, the former contained no detectable copper (<0.08%) unlike the latter with 3.8% by mass according to ICP-OES. Both brake pads are considered comparable to the 65 brake pads chemically analyzed by Hulskotte et al.,^47^ although the exact pad types (NAO or LM) represented in that study were not specified. The observed absence of the brake-associated elements Pb, Sb, or Ti in both our pads is consistent with the lower-bound concentrations reported by Hulskotte et al., highlighting the variability in elemental composition across different brake pad formulations.

Iron was the most abundant element by mass in the fine and coarse BWP_LM_ and BWP_NAO_ (Table 1). This finding agrees with other studies investigating BWP of similar sizes with unnamed friction material^48,49^ or LM^10,32,46,50–55^ and NAO^32,46,53–56^ pads in combination with cast iron discs.

The iron in our BWP was found to be spatially correlated with oxygen (Figures 1 and S8.3), suggesting iron oxide as the most abundant compound by mass. The 0.25 nm spacings from the lattice fringes in the high-resolution TEM images (Figure S8.7) imply the presence of magnetite (hkl 311) or maghemite (hkl 313) or both.^57^ The spacings of 0.27 and 0.26 nm were seemingly less frequent, implying the coexistence of hematite in lower abundance (hkl_hex_ 104 and 110, respectively). No goethite-typical spacings of 0.42 nm (hkl 101) were found. Magnetite,^43,52,55,58^ maghemite,^43,55^ and hematite^43,52,55,58^ have been reported before in airborne BWP from disc brakes. The iron in the brake disc and pads was found to be mainly elemental, suggesting temperature-related tribo-oxidation as a formation mechanism for the iron oxide.^26,52^ When calculating mass balances, not only the mass contributions of the brake pads and disc, but also the air should be considered.

Although the NAO pad contained only approximately 1% of iron, the corresponding airborne BWP were nonetheless iron-rich, with mass fractions of about 33%. We conclude that the airborne iron in case of NAO mainly originated from the brake disc and not the pad, consistent with previous findings.^26,48,51,52,59^ Brake discs made from gray cast iron have relatively high carbon mass fractions of approximately 3% and thus increased thermal diffusivity in comparison with, for example, steel.^60^ At the same time, the carbon results in a microstructure characterized by graphite flakes (Figure S8.10) which are associated with increased brittleness.^61^

The mass ratio of approximately 4 between barium and sulfur (Figures 1D and S8.5) suggests BaSO_4_ as the second most abundant compound in the fine and coarse BWP collected in this study. BaSO_4_ is commonly used as a filler in brake pads.^17^

The copper-rich micron-sized particles in BWP_NAO_ (Figure S8.12) have likely originated from the NAO brake pad as such particles were not found for BWP_LM_. Nevertheless, also the latter BWP show approximately 0.1% of submicron copper by mass, which is likely attributable to the brake disc.

The level of carbon was found to increase with decreasing particle size as shown in Figure 1D and Neukirchen et al.^46^ The observation may result from carbonaceous material coating particles of all sizes, with smaller particles appearing more affected due to their higher surface-to-mass ratio. The origin of this carbonaceous material is uncertain but may involve condensation of vaporized organics from the pad binder.

#### 4.2.3 Particle Formation Mechanism

Localized regions of elevated Al, Ca, Cu, Mg, and Si concentrations occasionally occur within individual BWP, suggesting that mechanical abrasion contributed to their formation. These mineral-rich inclusions appear to be embedded within a matrix that exhibits a surprisingly high degree of elemental homogeneity, comparable to a chocolate chip cookie. If the particles are formed during the mechanical fragmentation of brake pad or disc, we would expect individual BWP to be dominant in material attributable to one or the other. However, the coexistence of pad- and disc-associated elements was found in particles not only at the micro- but also at the nanoscale. Surprisingly, the observed elemental ratios at nanoscale were similar to the BWP bulk.

Other authors suggested that brake wear nanoparticles are formed upon evaporation and condensation of the friction material.^43,62^ Micron-sized brake particles, however, are believed to form during mechanical wear.^58,59,62^ We consider a mechanism that unifies both considerations to explain nano- as well as micron-sized BWP: the friction heat causes evaporation from the brake pad and disc; the gases nucleate into nanoparticles, explaining the spherelike shapes and highly mixed compositions (Figure 1). Eriksson and Jacobson^63^ described how small debris at the brake interface flows through a three-dimensional maze of shallow channels to form secondary plateaus as it occasionally gets jammed against more wear-resistant structures. We hypothesize that the small debris is composed of vapor-condensed nanoparticles which are compacted in secondary plateaus and eventually worn as flakelike particles with aerodynamic diameters exceeding 1 μm. In the cookie analogy, the dough represents the vapor-condensed nanoparticles, and the chips correspond to mechanically abraded fragments.

Analogous particle formation processes via rapid localized heating are documented in high-energy systems such as laser ablation^64,65^ and spark discharge.^66,67^ These studies report nanoparticles of sizes and morphologies that resemble those observed here, supporting the plausibility of a high-temperature volatilization–condensation pathway.

Maximum temperatures at the friction interface can exceed 1,000 °C, as observed by Sutter and Ranc^68^ for steel at spatial resolutions of approximately 100 μm. At smaller scales, particularly at the asperity or atomic level, even higher localized temperatures can be expected, potentially sufficient to induce iron evaporation. We observed significant formation of sub-10 nm iron nanoparticles by evaporation and nucleation above 1100 °C, where number concentration rapidly increased with evaporation temperature (Figure S9.1). This is consistent with the presence of spherelike iron oxide nanoparticles in our BWP, which are characteristic of nucleation from a highly supersaturated vapor, as well as with the relatively high CO_2_ emissions of 1.7 ± 0.15 mg/km/brake quantified by Hagino et al.^69^

Although hypothetical, the extreme power dissipation at the friction interface could trigger evaporation–condensation processes leading to particle emissions potentially relevant in both number and mass. Eriksson and Jacobson^63^ compared the heat dissipation during hard braking in a family car, about 80 TW/m^3^, to the output of 80 nuclear reactors concentrated within one cubic decimeter. Such brief yet extreme conditions may explain the formation of mixed-composition nanoparticles that compact into larger aggregates. This hypothesized mechanism is supported, but not proven, by the available evidence. Further work is needed to better constrain the thermal conditions and particle formation dynamics during braking.

### 4.3 Cellular and Acellular Assays

This study focused on oxidative stress and DNA damage responses as initial indicators of particle-induced toxicity. Other mechanistic pathways, including inflammatory, apoptosis, and fibrogenic responses, are also relevant to BWP toxicity but were beyond the scope of the present work.

BWP_LM_ and BWP_NAO_ caused significant ROS production, antioxidant depletion, and DNA strand breaks after 24 h exposure in a concentration-dependent manner. Consistent with the chemical characterization, no clear differences were observed between fine and coarse BWP. Interestingly, the toxicological differences between BWP_LM_ and BWP_NAO_ depended greatly on the end point. No clear differences between the two brake pads were observed for the cell viability, cellular ROS formation, and cellular GSH depletion. In contrast, BWP_NAO_ caused significantly more acellular antioxidant depletion and cellular DNA damage per particle mass than BWP_LM_. This apparent discrepancy may reflect differences between extracellular and intracellular oxidative environments as well as the specificity of the assays. Acellular tests quantify total oxidation potential, whereas cellular responses are modulated by antioxidant defenses, enzyme activity, and the nature of the reactive species involved.

The present work does not directly identify constituents in BWP that may cause oxidative stress and DNA damage, and it is important to note that the observed effects cannot be clearly attributed to any specific component. Organic constituents such as PAH’s were not investigated in this study, but their presence in BWP has been reported previously^28,29^ and may have contributed to the toxicological effects observed here. Nevertheless, we observe that quantities of transition metals differ between BWP_LM_ and BWP_NAO_, with copper being higher by mass in the latter. Parkin et al. looked at the effects of BWP with aerodynamic diameter 0.1–2.5 μm generated from different brake pad types at a concentration range of 12.5–100 μg/mL with an immortalized alveolar type-II cell line in submerged conditions.^32^ Similar to our study, Parkin et al. reported more oxidative stress and inflammation for BWP generated with NAO and ceramic pads, than for LM, and SM. Using a range of metal chelators, the authors demonstrated that NAO-derived copper can accumulate intracellularly and likely contribute to the observed effects. Figliuzzi et al. also used submerged conditions and found a positive correlation with four samples of fine BWP with increasing copper concentrations and cell viability, mitochondrial membrane potential change, and apoptosis in A549 cells.^31^ They used the highest concentration (100 μg/mL) to show altered expression of genes involved in apoptosis, DNA repair, inflammation and oxidative stress. Additionally, they generally found a positive correlation between copper content of brake-wear and ROS production in A549 cells. However, this did not hold true in the sample with the lowest amount of copper. They concluded that a reason for this may be the presence of unanalyzed heavy metals in the sample that also induce ROS production in a concentration-dependent manner. Furthermore, it has been shown that copper­(II) oxide nanoparticles can cause DNA damage in A549 and a bronchial cell line (BEAS-2B), whereas copper­(II) chloride does not.^70^ Although the toxicological impact of copper in BWP remains elusive, its role as an element of interest becomes clearer.

There is a limited number of studies evaluating genotoxicity of BWP and, to the best of our knowledge, no studies on comet assay end points. Melzi et al. used submerged conditions to show increased genotoxic effects in γH2AX and micronucleus assay by exposure to dust from unspecified brake pad linings (0.6 μm in mode size) at concentrations of 25–150 μg/mL in BEAS-2B cells.^30^ In a pilot study using blood samples from a single donor, Kazimirova et al. showed that BWP_LM_ with unspecified particle sizes could increase micronucleus formation at concentrations of 3–75 μg/cm^2^ in human lymphocytes.^71^ The present study has several lines of evidence indicating that the observed genotoxic effect is related to oxidative stress: (i) increased ROS production and glutathione depletion, (ii) induction of DNA strand breaks blunted by NAC, and (iii) the increase in Fpg-sensitive sites. The latter is considered to be caused by oxidation of nucleobases (e.g., oxidation of guanine to 8-oxoguanine), whereas DNA strand breaks may be generated by ROS (e.g., hydroxyl radicals from degradation of hydrogen peroxide). However, as cellular redox chemistry is complex, not all ROS species contribute equally to DNA damage, and the oxidants detected by the DCFH-DA assay may differ from those that cause strand breaks. Likewise, intracellular antioxidant defenses including glutathione and enzymatic scavengers further modulate the extent of oxidative genotoxicity. This complexity may explain why the degree of DNA damage did not directly mirror changes in ROS formation or GSH depletion.

Puisney et al.^33^ have used submerged condition and found that nano- and microscale BWP_LM_ show increases in ROS production and the pro-inflammatory cytokine interleukin 6 (IL-6) in human lung adenocarcinoma (Calu-3) cells in a concentration-dependent manner from 3–300 μg/mL. However, a later study from the same group showed only a modest effect on secretion of the proinflammatory cytokine tumor necrosis factor (TNF), whereas proinflammatory cytokines IL-6 and IL-8 levels were unaltered.^72^ Trečiokaitė et al. showed reduced cell viability and increased ROS production when looking at BWP generated with ceramic pads and a steel disc at concentrations of 10–80 μg/mL in A549 cells.^73^

Most studies on BWP toxicity have used either submerged conditions, like the present study. It is also possible to use more advanced exposure systems such as air–liquid interface (ALI), which is typically considered to be more realistic in terms of exposure, although it is not clear whether it produces markedly different results compared to exposure in submerged conditions.^74^ Using an ALI system consisting of A549 and primary immune cells, Barosova et al. showed that nonairborne BWP_NAO_ produced a significant, concentration-dependent increase in cell death and a 2- to 4-fold significant increase in IL-8, while no IL-8 effects were observed for nonairborne BWP_LM_. In contrast to our results, they find no effects on GSH levels with either BWP_LM_ or BWP_NAO_ samples.^34^ Gasser et al. used an ALI exposure system with A549 cells exposed to air emitted from the disc brake of a Renault Laguna 2.0. They found significant negative correlations between the density of the tight junction protein occludin and the concentration of metals (iron, copper, manganese).^75^

Concentrations of 20 and 100 μg/mL as used in our study are likely higher than real-world exposure levels of BWP, though they may represent worst-case scenarios. On the other hand, concentrations of 0.8 and 4 μg/mL may better reflect ambient conditions.^34^ We selected these concentrations to align with the existing literature and to simulate acute effects. As BWP may have the potential to accumulate in tissues,^72^ future studies should focus on chronic exposure to better understand the effects under real-world conditions. A549 cells were selected as a model of the human lung epithelium, commonly used for assessing oxidative stress and certain forms of DNA damage. Their widespread use in toxicological research enables comparison with existing studies. Nonetheless, future investigations should incorporate other cell lines to evaluate additional genotoxic (e.g., micronucleus assay) and nongenotoxic (e.g., inflammation) end points.

In conclusion, our results show that airborne BWP are a concern for lung health. The higher toxic potency (especially in acellular antioxidant depletion and DNA damage) of BWP_NAO_ may be related to the chemical composition, e.g., copper which is an order of magnitude more abundant in BWP_NAO_. However, our findings suggest that the level of copper alone does not fully account for the observed effects since both BWP_LM_ and BWP_NAO_ elicited toxic responses. Further research is needed to identify the most toxic constituents of automotive disc brakes and to develop alternatives with improved biocompatibility.

## Supplementary Material



## Notes

Additional methodological details, microscopic images, and acellular and cellular toxicological results (PDF)
