# Supplementary material for: Physicochemical and Toxicological Characterization of Airborne Brake Wear Particles Reveals Oxidative Stress–Mediated DNA Damage
Source: Environ Sci Technol. 2026 Feb 3;60(6):4955–65. doi: 10.1021/acs.est.5c10783 (PMC12918519; doi:10.1021/acs.est.5c10783)
Supplement: Supplementary file 1 [file es5c10783_si_001.pdf]

Supporting Information

Physicochemical and Toxicological Characterization of Airborne Brake Wear Particles Reveals Oxidative Stress–Mediated DNA Damage

Samuel Hyman[1,2,+,*], Siriel Saladin[3,*], Yurii Tsybrii[4,*], Oleksii Nosko[4,+], Matthew Williams[3], Alexander Zherebker[3], Kelvin Risby[5], David Topping[1,+], Adam Boies[5], Chiara Giorio[3,+], Martin Roursgaard[2], Peter Møller[2,+]

[1] Department of Earth and Environmental Science, Centre for Atmospheric Science, School of Natural Sciences, The University of Manchester, Manchester M13 9PL, United Kingdom

[2] Section of Environmental Health, Department of Public Health, University of Copenhagen, Copenhagen K 1014, Denmark

[3] Yusuf Hamied Department of Chemistry, University of Cambridge, Cambridge CB2 1EW, United Kingdom

[4] Faculty of Mechanical Engineering and Ship Technology, Gdansk University of Technology, Gdansk 80-233, Poland

[5] Department of Engineering, University of Cambridge, Cambridge CB2 1PZ, United Kingdom

*These authors contributed equally to this work.
[+] Corresponding authors

Number of pages: 28
Number of figures: 18
Number of tables: 5

Section S1: Overview

Table S1.1: Overview of all collected samples and conducted experiments.

Assay	Brake Disc	LM pad	NAO pad	BWP_{LM}			BWP_{NAO}		
				ELPI+ stages					
				**#2–9** (16–940 nm)	**#10–11** (0.94–2.5 µm)	**#12–14** (2.5–10 µm)	**#2–9** (16–940 nm)	**#10–11** (0.94–2.5 µm)	**#12–14** (2.5–10 µm)
DLS	-	-	-	-	Yes	Yes	-	Yes	Yes
CHN	Yes	Yes	Yes	-	Yes	Yes	-	Yes	Yes
ICP-OES	Yes	Yes	Yes	-	Yes	Yes	-	Yes	Yes
SEM	Yes	Yes	Yes	Yes	Yes	Yes	Yes	Yes	Yes
TEM	-	-	-	-	-	Yes	-	-	Yes
Acellular antioxidants	-	-	-	-	Yes	Yes	-	Yes	Yes
Cellular WST-1	-	-	-	-	Yes	Yes	-	Yes	Yes
Cellular LDH	-	-	-	-	Yes	Yes	-	Yes	Yes
Cellular GSH	-	-	-	-	Yes	Yes	-	Yes	Yes
Cellular ROS	-	-	-	-	Yes	Yes	-	Yes	Yes
Cellular DNA	-	-	-	-	Yes	Yes	-	Yes	Yes

DLS Dynamic light scattering
CHN Carbon, hydrogen, nitrogen analysis
ICP-OES Inductively coupled plasma optical emission spectroscopy
SEM Scanning electron microscopy
TEM Transmission electron microscopy
WST-1 Water-soluble tetrazolium salt 1 assay
LDH Lactate dehydrogenase assay
GSH Glutathione
ROS Reactive oxygen species
DNA Deoxyribonucleic acid

Section S2: Generation and collection of wear particles

Method S2.1: Pin-on-disc experimental setup

Wear particles were generated using a pin-on-disc test setup based on an Rtec MFT-5000 tribometer. The friction pair consisted of a pin specimen and a disc specimen, each simulating a brake pad and a brake disc, respectively. The pin specimen was a cylinder with a diameter of 10 mm and a thickness of 8 mm. The disc specimen had a diameter of 58 mm and a thickness of 30 mm. During the friction test, the disc specimen was rotated, while the pin specimen was pressed against the disc specimen, with a friction radius of 21 mm. The temperature inside the pin specimen was measured with a thermocouple at a distance of 1 mm from the friction surface.

Method S2.2: Friction materials

The pin and disc specimens were cut from the respective brake pads and discs intended for a passenger car and available on the EU market. The pin specimens were cut using a CNC machine. There were two types of brake pads: LM and NAO. The NAO brake pad was classified based on the manufacturer's specification, which designates this formulation as NAO. Elemental analysis showed a low iron mass fraction (1.1%) for the NAO pad, compared with a higher iron mass fraction (22%) for the LM pad. We note that brake pad classifications are not standardized. The disc specimens were cut by water jet from a new passenger-car gray cast iron brake disc. The pin and disc specimens were cut without altering the original friction surfaces. The disc surfaces were cleaned with a rust-removal solution, and both the pin and disc specimens underwent a 30-minute burnishing procedure under conditions similar to those of the main experiments. After burnishing, the disc surfaces were cleaned with pressurized air to remove any remaining residues before the main tests. The pin and disc specimens were replaced with fresh samples between test runs.

Method S2.3: Wear particle collection system

The friction pair was isolated from the environment by a sealed chamber. A compressor–receiver system provided a stable airflow of 12 L/min at the inlet of the chamber. The inlet air was filtered and dried using a TSI Filtered Air Supply 3074B with a nominal filtering efficiency of 99.99995% at 0.1 µm. The friction between the pin and disc specimens led to the generation of wear particles. The airborne fraction of the wear particles was transported to the chamber outlet by the airflow. Thereby, the chamber ensured that the airflow in it and at its outlet contained only wear particles.

The collection of airborne wear particles was performed with an electrical low pressure impactor (ELPI+) from Dekati. This instrument sampled the air in the chamber in the vicinity of the friction pair with a rate of 10 L/min. The sampled air passed then through the impactor, where the wear particles were classified by size according to their aerodynamic diameters and collected on substrates. ELPI+ has 14 stages numbered #15 down to #2 with particle cutoff diameters (50% collection efficiency) of 10, 5.3, 3.6, 2.5, 1.6, 0.94, 0.60, 0.38, 0.25, 0.15, 0.094, 0.054, 0.030, and 0.016 µm. Stage #15 collects thus particles greater than 10 µm and is used as a pre-filter. Stages #12, #13, #14 collect 2.5–10 µm particles denoted as coarse. Stages #11 and #10 collect 0.94–2.5 µm particles denoted as fine. The substrates used were round aluminum foils with a diameter of 25 mm and a thickness of 0.1 mm. The aerosol facing surface of each substrate was dry and clean (not greased).

Method S2.4: Friction conditions

Each friction test consisted of a running-in phase and a particle collection phase. The running-in phase, which lasted 30 min, was intended to reach steady roughness parameters of the friction surfaces and to increase the apparent friction contact area to 100%. No wear particles were collected on the ELPI+ substrates

during the running-in phase. The particle collection phase lasted 12 or 18 h (Table S7.1). During both phases, the disc specimen rotated with an angular speed of 910 revolutions per minute, which corresponded to a sliding speed of 2 m/s at the friction radius. In a passenger car, the ratio between the effective radius of the brake disc and the radius of the wheel is about 0.4, so the mentioned sliding speed between the pin and disc specimens corresponds to the average sliding speed in the brake when the car brakes uniformly from an initial speed of 36 km/h to zero. The pin specimen was pressed against the disc specimen with a normal force of 39 N, providing a contact pressure of 0.5 MPa. The ambient temperature was 25 ± 2 °C. The steady temperature in the pin specimen did not exceed 100 °C. Thereby, the provided values of the sliding speed, contact pressure and temperature were characteristic of the mechanical brake of a passenger car operated in mild braking mode, i.e., where both the sliding speed and contact pressure are not high.

Method S2.5: Cleaning procedures for particle collection

Between the running-in and particle collection phases of each friction test, the chamber and the components of the tribometer inside the chamber were cleaned with 99.9% isopropanol. The mechanical components of the ELPI+ cascade impactor were cleaned in an ultrasonic bath with 99.9% isopropanol solution for 30 min. The installation of the substrates onto the ELPI+ impactor plates before the particle collection phase and their deinstallation after the friction test were performed by the operator wearing a medical mask and nitrile gloves.

Section S3: Temperature and particle size

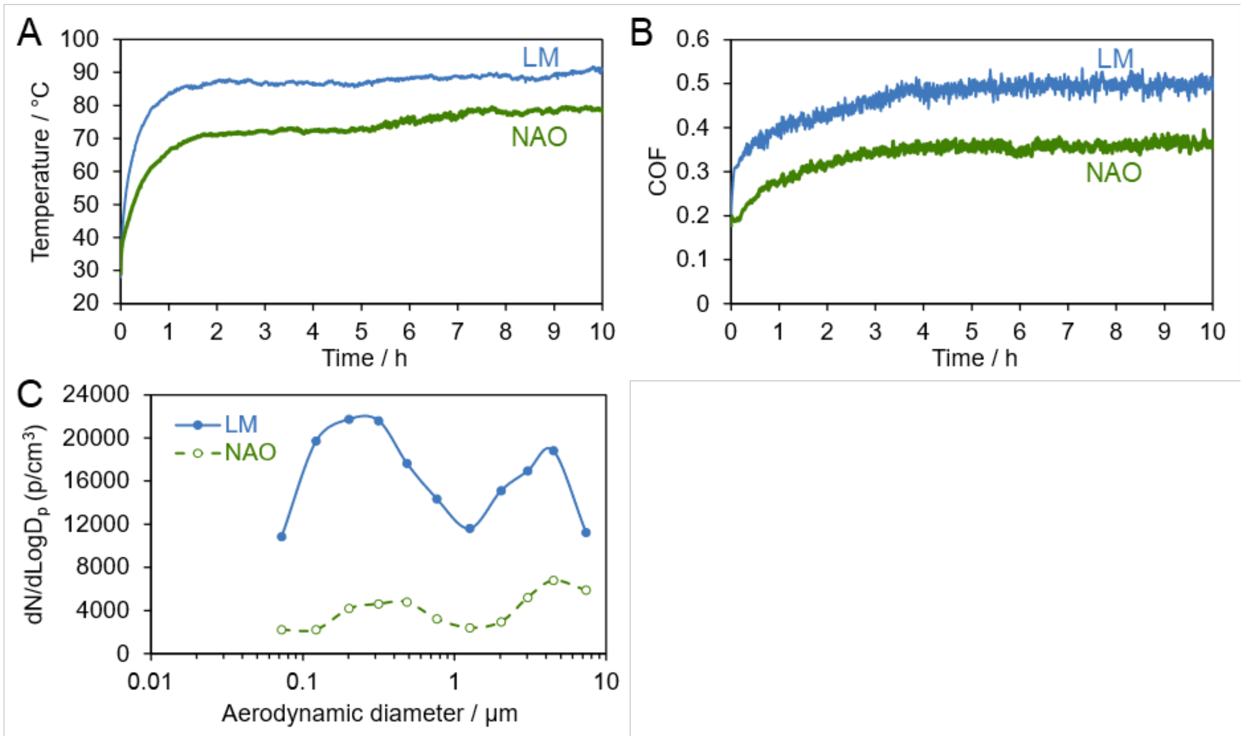

Figure S3.1: (A) Typical brake pad temperature profiles as measured with a thermocouple during the collection of airborne BWP_{LM} and BWP_{NAO}. (B) Measured coefficients of friction (COF) for both brake pads. (C) Typical particle size distributions of airborne BWP_{LM} and BWP_{NAO} as measured by the ELPI+.

Section S4: Chemical characterization

Method S4.1: Sample preparation for chemical analysis

The brake pads were cryomilled (Retsch, liquid nitrogen) for 10 min at 5 Hz and then 30 min at 20 Hz in a 50 mL stainless steel jar with one 25 mm stainless steel ball. The brake disc was pulverized using a lathe. The jar and lathe were carefully cleaned with water and ethanol prior to each run. The BWP were already in powder form, so no further preparation was performed for chemical analysis. The samples in powder form were used for CHN and ICP-OES analysis as well as microscopy in case of BWP.

Method S4.2: Dynamic light scattering (DLS)

BWP dispersed in ultrapure water (resistivity ≥ 18.2 MΩ·cm at 25 °C) at 10 µg/mL were studied with DLS using a Zetasizer Nano ZSP from Malvern.

Method S4.3: CHN analysis

A CE-440 Elemental Analyser from Exeter Analytical was used for quantification of C, H, and N (2 mg per sample, duplicates). The combustion and reduction tube temperatures were set to 975 °C and 600 °C, respectively.

Method S4.4: Inductively coupled plasma optical emission spectroscopy (ICP-OES)

For every sample, BWP (2 mg) or brake pad/disc powder (15 mg) were taken in triplicates and digested for 1 h with 0.9 mL hydrochloric acid (37%, 07102, Honeywell) at 85 °C in an ultrasound bath. The samples were supplemented with 2.1 mL nitric acid (69%, 450043X, VWR) and again digested for 1 h under the same conditions. The mixtures were filtered (0.2 µm, PTFE), diluted to 0.1 M HCl and 0.6 M HNO_3 in ultrapure water (resistivity ≥ 18.2 MΩ·cm at 25 °C), and analyzed with an iCAP 7400 Duo ICP Spectrometer from Thermo Fisher Scientific. The multielement standard 6 (43843, Supelco) was used for external calibration with nine points covering a concentration range of 0.01–10 mg/L. Coefficient of determination was $R^2 > 0.99$ for all 23 elements. The HCl and HNO_3 concentrations of the calibration standard were adapted to match the matrix of the samples. A plasma RF power of 1150 W, coolant gas flow of 12 L/min, nebulizer gas flow of 0.40 L/min, peristaltic pump speed of 60 rpm, sample delay time of 70 seconds, and sample wash time of 60 seconds were used.

Not all elements dissolve equally well during acid digestion, which may introduce a bias if some constituents remain undigested and thereby lead to underestimation or false negatives. To assess this, we isolated the undigested residue and analyzed it by SEM-EDS. The residue contained silicate-rich particles, which are expected to remain undigested without the use of hydrofluoric acid. Consequently, we used the quantitative SEM-EDS data instead of ICP-OES to report the mass fractions of silicon. All metals detected by SEM-EDS were also identified by ICP-OES in comparable quantities.

Method S4.5: Scanning electron microscopy (SEM)

The SEM images were captured with a TESCAN CLARA-2 scanning electron microscope equipped with an Oxford Instruments X-maxN 80 EDS system. EDS maps were acquired at 10 keV. The particles in powder form were transferred to carbon tape, while the BWP on the ELPI+ stages 2 to 9 were kept on the ungreased aluminum substrates. Pins of unworn and worn brake pad and disc were analyzed without further sample preparation. The elemental composition was assessed in triplicates based on different regions of the pins or densely packed particles using field of views of at least 50 µm. No elements were excluded from the mass balance in case of BWP from stages 10 to 14. These particles were studied on a carbon substrate and thus it cannot be ruled out that background carbon is detected, which would lead to an overestimation of carbon and underestimation of the other elements. Unlike stages 10 to 14, the aluminum substrates of stages

2 to 9 were less densely packed with BWP and aluminum was therefore excluded from the mass balance. No sputter coating was used for any sample.

Method S4.6: Transmission electron microscopy (TEM)

The TEM and scanning transmission electron microscopy (STEM) images, and EDS data were obtained using a Thermo Scientific Talos F200X G2 operated at 200 kV. The TEM images were recorded using a Ceta 16k CMOS camera. STEM images were collected using a Fischione high-angle annular dark-field (HAADF) detector at a camera length of 98 mm and a bright field (BF) detector. STEM-EDS spectra and maps were acquired using the Super-X EDS detector system which consists of four windowless silicon drift detectors. The samples were prepared by pipetting 2.5 μL of an ultrasonicated ethanol suspension onto continuous carbon 300 mesh Ni grids (EM Resolutions). A low background double tilt sample holder with an aluminum spacer and molybdenum clamp was used for the analysis. Spacings from the lattice fringes of individual particles were measured with TEM imaging.

Section S5: Acellular antioxidant depletion

Method S5.1: General

Ultrapure water (resistivity ≥ 18.2 MΩ·cm at 25 °C) was prepared using a Milli-Q® Advantage A10 water purification system from Merck. We avoided using glass or metalware throughout the procedure. All plastic equipment was washed with 2% nitric acid (diluted from 69%; ARISTAR®), LC-MS grade methanol (Fisher Scientific), and ultrapure water.

Method S5.2: Surrogate epithelial lung fluid (SELF)

The SELF used in this study was prepared in accordance with SELF-e from Shahpoury et al.[1] with minor adaptations as detailed in Table 5.2.1. The inorganic portion was prepared as a batch using phosphate buffered saline tablets (Sigma-Aldrich) and Na_2SO_4. Chelex 100 in sodium form was used to mitigate metal ion contamination prior to addition of Mg and Ca salts. The organics were added prior to experimentation, including uric acid, 1,2-dihexadecanoyl-sn-glycero-3-phosphocholine (DPPC), bovine serum albumin, and glycine. The SELF was adjusted with hydrochloric acid to obtain a physiological pH of 7.4 ± 0.1. The antioxidants glutathione (GSH), cysteine (CYS), and ascorbic acid (AA) were added upon commencement of the experiment to minimize depletion before time zero.

Table 5.2.1: Composition of SELF used in this study, adapted from Shahpoury et al.[1]

Compound	Concentration / (mg/L)
NaCl	8065
KCl	201
Na_2HPO_4	1150
KH_2PO_4	200
Na_2SO_4	72
$CaCl_2$	256
$MgCl_2$	200
Uric acid	16
DPPC	100
Albumin	260
Glycine	376
AA	35
GSH	62
CYS	24

Method S5.3: Sample preparation

4 mL of SELF were transferred to 8 mL polystyrene round-bottomed tubes (Non-pyrogenic; Falcon) and incubated at 37 °C. Each brake wear suspension was added to the SELF at three concentrations: 4, 20, and 100 µg/mL while maintaining a total volume of 4 mL. A control tube of SELF without any particulate matter served as a negative control. The tubes were then supplemented with the antioxidants GSH, CYS, and AA. Aliquots of 300 µL were taken and transferred to 2 mL centrifuge tubes containing 100 µL of 100 mM *N*-ethylmaleimide (NEM). The centrifuge tubes were vortexed for 2 min to allow for scavenging of free thiols. Subsequently, 200 µL of a solution with 2% sulfosalicylic acid (SSA) and 2 mM ethylenediaminetetraacetic acid (EDTA) was added to each aliquot and vortexed for a further minute. EDTA was used to chelate metal ions and SSA was used to precipitate albumin and reduce the pH to stabilize AA. Aliquots were centrifuged

(10,000 g, 6 min) and 4 µL of supernatant were transferred to plastic LC-MS vials and diluted with ultrapure water with 0.1% formic acid to give a 1 mL solution. LC-MS samples were vortexed and stored at 4 °C before analysis. Further aliquots were collected 40, 80, 120, and 180 min after addition of brake wear particles to characterize the kinetic profile of antioxidant depletion and glutathione disulfide (GSSG) accumulation.

Method S5.4: Instrument parameters

Liquid chromatography mass spectrometry (LC-MS) was performed on an Exion LC unit coupled with a triple quadrupole mass spectrometer (QTRAP 5500+) both from SCIEX. Separation of analytes was achieved with a reversed phase column (ZORBAX Eclipse Plus C18, 3 mm, 150 mm, 3.5-micron) from Agilent. The mobile phase A consisted of ultrapure water with 0.1% formic acid and mobile phase B consisted of LC-MS grade acetonitrile containing 0.1% formic acid. The mobile phase flow rate was set at 0.4 mL/min with a gradient as outlined in Table S5.4.1. Electrospray ionization was used in negative mode for the analysis of AA and positive mode for GSH, GSSG, and CYS analytes. The autosampler temperature was set at 4 °C with an injection volume of 10 µL.

Table S5.4.1: Gradient used for liquid chromatography in the acellular antioxidant depletion assay.

Time / min	Phase A (water) / %	Phase B (acetonitrile) / %
0	99	1
0.3	99	1
0.9	50	50
7.5	50	50
8.0	5	95
11.0	5	95
12.0	99	1
15.0	99	1

Table S5.4.2 presents the detection parameters of the analytes in the multiple reaction monitoring mode. Two daughter ions were screened for each respective analyte with the strongest peak being used for quantitative analysis and the other as a qualitative check to confirm the analyte identity. The following MS/MS parameters were used: scheduled ionization from 1.8 to 15 min, ion spray voltage of 2.5 kV, source temperature of 500 °C, ion source gas flow rates of 50 arbitrary units (au), curtain gas flow rate of 35 au, and collision gas flow rate set to 9 au. Quantification of analyte peaks was achieved through a six-point external calibration ($R^2 > 0.99$) ranging from 1 to 100 µg/L. The aliquots from the last time points were injected in triplicates. Blanks were run between each sample set to mitigate any analyte carry-over. Chromatographic data analysis was performed using OS-MQ Software (SCIEX).

Table S5.4.2: LC-MS/MS parameters for detection of target analytes.

Analyte	Parent ion *m/z*	Daughter ion *m/z*	Retention time (min)	Collision energy (V)	Declustering potential (V)	Entrance potential (V)	Cell exit potential (V)
AA [quant.]	175	115	2.27	-18	-9	-10	-11
AA [qual.]	175	71	2.27	-25	-9	-10	-11
GSH-NEM [quant.]	433	304	3.27	21	110	-10	22
GSH-NEM [qual.]	433	201	3.27	31	110	-10	16
GSSG [quant.]	247	484	3.27	27	140	-10	14
GSSG [qual.]	247	231	3.27	47	140	-10	20
CYS-NEM [quant.]	613	158	3.30	31	80	-10	12
CYS-NEM [qual.]	613	126	3.30	35	80	-10	14

Section S6: Cellular assays

Method S6.1: Cell Culture and exposure

A549 cells (American Type Culture Collection, Manassas, Virginia, USA) were cultured in F12 Ham Nutrient mix supplemented with 10% foetal bovine serum (FBS), 1% L-glutamine, and 1% penicillin/streptomycin (all from Gibco) at 37 °C and 5% CO_2. The cells were cultured in full cell culture medium for 24 h before exposure to BWP or positive controls for 3 or 24 h using the Nanogenotox Dispersion Protocol.[2] The negative control groups in all assays consist of cells and cell culture medium only. All exposures have been repeated in at least three independent experiments on separate days. To make BWP stock solutions, dry BWP were added to hydrophobic plastic vials and then Water for Injection for Cell Culture was added. Before use, vials were inverted, sonicated and vortexed. The cells were exposed to BWP particle concentrations between 0.8 and 100 µg/mL, which corresponds to surface concentrations of 0.5–62.5 µg/cm^2 in 96-well plate assays (200 µL per well), or 0.42–52.6 µg/cm^2 in 24-well plate assays (1 mL per well).

Method S6.2: Cytotoxicity

Cytotoxicity was assessed by a combination of the intracellular production of formazan from water-soluble tetrazolium 1 (WST-1) by dehydrogenase enzymes and lactate dehydrogenase (LDH) activity in the cell medium (i.e. indicator of damage to the cell membrane). Cells (50,000 per well) were plated in transparent 96-well plates and exposed for 24 h to BWP or 1% Triton™ X-100 (positive control). After the exposure period, 100 µL of the supernatant was transferred to a new transparent 96-well plate, and 100 µL of LDH working solution (Lactate Dehydrogenase Activity, Roche Diagnostics) was added to each well. The plate was covered with aluminum foil and left at room temperature for 15 min. The LDH activity was assessed by spectrophotometric analysis, using absorbance wavelengths at 500 nm and 630 nm as the reference wavelength (Multiskan FC Microplate Photometer, ThermoFisher). For the measurement of cellular dehydrogenase activity, all cell culture medium was removed from the wells and 100 µL WST-1 reagent (10%) in fresh cell culture medium was added. The cells were incubated for 1 h before measurement at a wavelength of 500 nm and 630 nm as reference wavelength.

Method S6.3: Intracellular GSH level

Cells (50,000 per well) were plated in 96-well black plates 24 h before exposure. The cells were exposed to BWP for 24 h in complete cell culture medium. Positive controls included 0.75 mM diethyl maleate (DEM) and 50 µM buthionine sulfoximine (BSO). DEM directly reacts with GSH, leading to rapid depletion, while BSO inhibits γ-glutamylcysteine synthetase, preventing GSH synthesis. ThioGlo-1 (Covalent Technologies, Inc., Walnut Creek, CA, USA) stock solution was diluted to 10 µM as a working solution for further dilutions in each experiment. The supernatant was removed and 100 µL of ThioGlo-1 working solution was added to each well. The plates were left at room temperature for approximately 5 min, followed by measurement of fluorescence (λ_{ex} = 355 nm; λ_{em} = 460 nm). The intracellular glutathione (GSH) concentration was calculated according to a GSH standard curve ranging from 0.125 to 16 µM and reported as nmol/10^6 plated cells.

Method S6.4: Intracellular ROS production

Intracellular ROS production was assessed by 2',7'-dihydrofluorescein diacetate (DCFH-DA) assay. Cells (50,000 per well) were seeded in black 96-well plates 24 h before exposure. At the day of experiment, cell culture medium was replaced with Hank's balanced salt solution (Gibco) containing DCFH-DA probe (10 µM). After 15 min of incubation, the cells were washed once with 200 µL Hank's balanced salt solution

to remove the extracellular probe. The cells were exposed for 3 h in cell culture medium. H_2O_2 (100–500 µM) was used as positive control. The fluorescence signal was measured at an excitation wavelength of 490 nm and an emission wavelength of 520 nm.

Method S6.5: DNA damage (Comet Assay)

Levels of DNA strand breaks were assessed by the standard comet assay. Cells were seeded in transparent 24-well plates at a density of 250,000 cells/well. After 24 h, the cell culture medium was removed, and the cells were exposed to BWP for 24 h (main experiment). In a separate experiment, the cell culture medium was supplemented with NAC 10 mM at the start of the 24 h exposure period. After the exposure, single cell suspensions were obtained by discarding the cell medium, washing the cells in 1 mL PBS, followed by trypsinization (i.e. 150 µL trypsin was added to each well and the reaction was stopped by adding 350 µL full cell culture medium after 5 min incubation at 37 °C and 5% CO_2).

100 µL cell suspension was mixed with 600 µL of 0.75% agarose and 120 µL was applied to GelBond films in duplicates. Positive controls were created by submerging selected gel bonds in baths of either 10, 50, or 100 µM H_2O_2 for 5 min, which were subsequently rinsed in cold phosphate buffered saline (PBS). Then, the cells were lysed for 1 h in lysis solution (2.5 M NaCl, 0.1 M Na_2EDTA, 10 mM Tris, 1% Triton X-100, pH 10) at 4 °C. Then for both versions of the comet assay, gels were placed in electrophoresis buffer (1 mM Na_2EDTA and 300 mM NaOH; with recycling of the solution) for 40 min at 4 °C, followed by 25 min electrophoresis at 300 mA and 20 V (0.83 V/cm from cathode to anode; measured voltage across the electrophoresis tank platform is 12.8 V or 0.53 V/cm).[3] The GelBond films were then rinsed with neutralizing buffer (0.4 M Tris) for 15 min, and left in 96% ethanol overnight. After drying, the samples were cut and stained with YOYO-1 (Thermo Fisher Scientific). Each gel was blindly scored under a fluorescence microscope at 40x magnification, where 100 cells from each gel were scored using a five-class scoring system (200 comets in total; score range between 0 and 100 arbitrary units (a.u.)).[4] The visual score in arbitrary units was converted to lesions/10^6 base pair by calibration with an investigator-specific calibration curve on gamma radiation.[5]

No general threshold for cytotoxicity was found at which the test results of the comet assay were flawed. A 25% increased cytotoxicity compared to the concurrent negative control is considered to be a good starting value to avoid false positive comet assay results, although even 50% cytotoxicity does not seem to affect background levels of DNA strand breaks.[6]

Levels of oxidatively damaged DNA were analyzed in a separate experiment, using the formamidopyrimidine DNA glycosylase Fpg-linked comet assay. For this version of the comet assay, slides with gel-embedded nucleoids were washed three times in enzyme buffer after the lysis step (5 min per wash). Following this, 60 µL Fpg (1 mg/mL, NorGeno-Tech, Norway) or enzyme buffer (40 mM HEPES, 0.1 M KCl, 0.5 mM Na_2EDTA, 0.2 mg/mL bovine serum albumin) were applied onto the gels and covered with a coverslip. The slides were incubated at 37 °C for 45 min. For the Fpg-linked comet assay, H_2O_2-exposed A549 cells were used as positive controls, and THP-1 monocytic cells exposed to potassium bromate (4.5 mM) served as controls for the enzyme treatment. The latter has been validated in multi-laboratory ring trials and it is considered sufficient information to assess the reliability of the Fpg-modified comet assay.[7,8] The results from the Fpg-linked comet assay are reported as DNA strand breaks and Fpg-sensitive sites (i.e. difference in DNA migration between slides treated with Fpg and enzyme buffer). The Fpg-sensitive sites encompass 8-oxoguanine and certain ring-opened purine bases.

Method S6.6: Interferences between particles and biomarker assays

Dedicated interference controls with BWP were not included in the present study. However, the selected assays have previously been validated for their robustness against technical flaws due to certain

nanomaterials ability to bind to probes or shielding of fluorescence light. These issues are typically considered to be related to the large surface area of nanomaterials. We have used the WST-1 assay because it is considered to have little interference with particles, based on observations on carbon nanotubes[9] and other nanomaterials[10]. The LDH assay has an inherent risk of bias because the enzyme leaks through damaged membranes to the particle-containing cell culture medium, which may decrease LDH activity as observed in studies on certain types of nanotubes[11]. However, tests on nanosized carbon black (Printex 90) at concentrations up to 100 µg/mL have not shown effect on LDH activity in cell culture medium and lyzed cell medium, although there was a slight decrease using pure LDH enzyme (approximately 20%).[12] In addition, potential fluorescence-shielding effects were evaluated and found not to bias comet-assay results.[13] Although potential assay interference by brake wear particles cannot be ruled out, it is considered unlikely based on insights from these validation studies.

Methods S6.7. Cellular internalization

It is well known that A549 cells internalize particulate matter. It has been demonstrated by fluorescence microscopy[14] and thermogravimetric-mass spectrometry (TGA-MS)[15–17] analysis. Biologically effective doses (based on internalization rates) is essential information for dosimetry in studies that specifically investigate differences in the toxic potency of nanoparticles. However, BWP are complex mixtures of particles with different sizes and chemical constituents, where the biologically effective dose cannot be described only by the mass of internalized particles.

Section S7: Collected masses of wear particles

In order to collect a sufficient amount of wear particles, several friction tests were performed for each of the brake pad materials (LM and NAO) as outlined in Table S7.1. The wear particles collected on substrate #15, which were specified as greater than 10 µm, were not included in the subsequent studies. Substrates #10 to #14 carried sufficient particle masses to be pooled (Figure S7.2A) unlike substrates #2 to #9 (Figure S7.2B). The substrates #2 to #9 were therefore not used for the toxicological assays. On average, approximately 65 mg BWP_{LM} and 45 mg BWP_{NAO} of coarse wear particles (substrates #12, #13, #14) and 22 mg BWP_{LM} and 12 mg BWP_{NAO} of fine wear particles (substrates #10 and #11) were collected during a single 18 h test. The wear particles collected on the corresponding substrates were pooled into a glass vial.

Table S7.1: Masses of the collected brake wear particles per ELPI+ stage. The particles were collected, pooled in glass vials, and then used for all experiments in this study. The particles did not strongly adhere to the aluminum substrates, allowing us to transfer approximately 90% of the collected particle mass into the glass vials by simple pouring.

**Brake pad**	**Test duration / h**	**Collected mass / mg**						
		**#9** (0.60–0.94 µm)	**#10** (0.94–1.6 µm)	**#11** (1.6–2.5 µm)	**#12** (2.5–3.6 µm)	**#13** (3.6–5.3 µm)	**#14** (5.3–10 µm)	**#15** (> 10 µm)
LM	12	0.5	2.8	8.6	14	18.4	4	0.75
	12	0.9	4.6	12.7	19.1	24.5	9.4	14.3
	18	0.8	4	11.9	21.1	30.2	10	23
	18	2.9	9.5	18.6	26	32.5	10.2	19.9
Total		5.1	73		219			57.95
**In glass vial**		-	**64**		**188**			-
NAO	12	0.6	1.6	6	12.1	16.4	7	7.8
	18	0.3	0.8	2.7	3.4	3.5	1.7	1.4
	18	0.9	3.2	8.6	16	21.3	8.3	8.7
	18	1.2	4.3	12.4	21.6	29.5	11	15
	18	0.7	1.5	5.3	9.7	12	5.5	4.4
Total		3.7	55		217			37.3
**In glass vial**		-	**52**		**187**			-

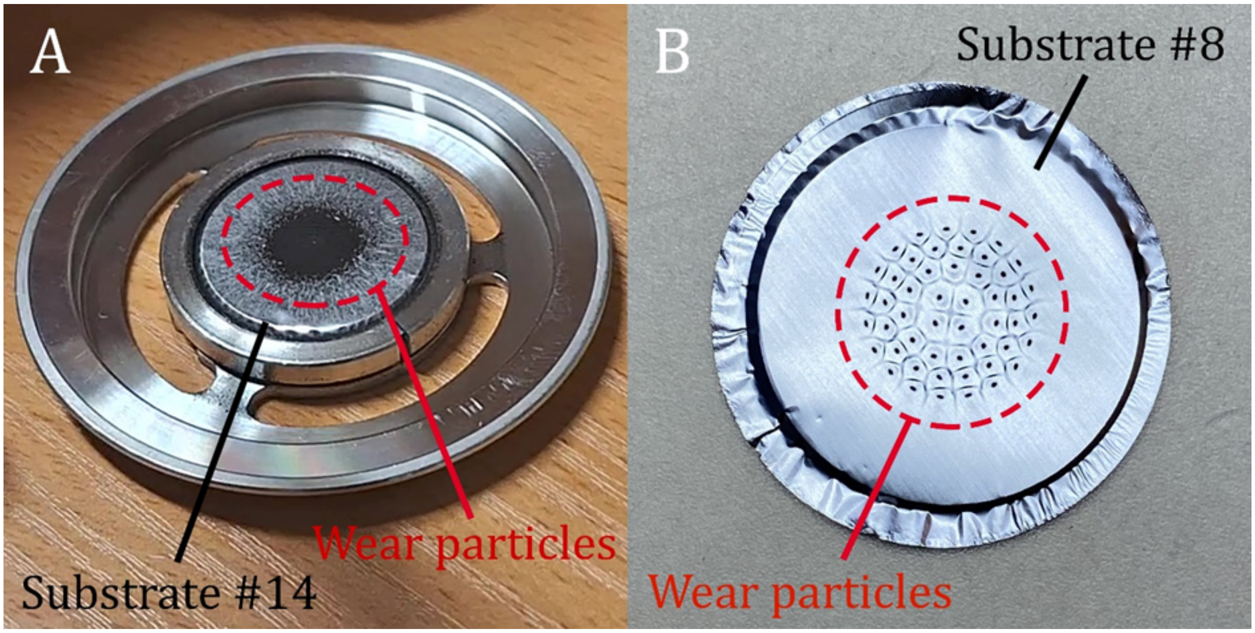

Figure S7.2: Substrates #10–14 from the ELPI+ carried a considerable amount of brake wear particles (A). In contrast, substrates up to and including #9 contained a scarce amount of wear particles (B). The particles on substrates #2 to #9 could not be quantitatively recovered given the low masses.

Section S8: Microscopy

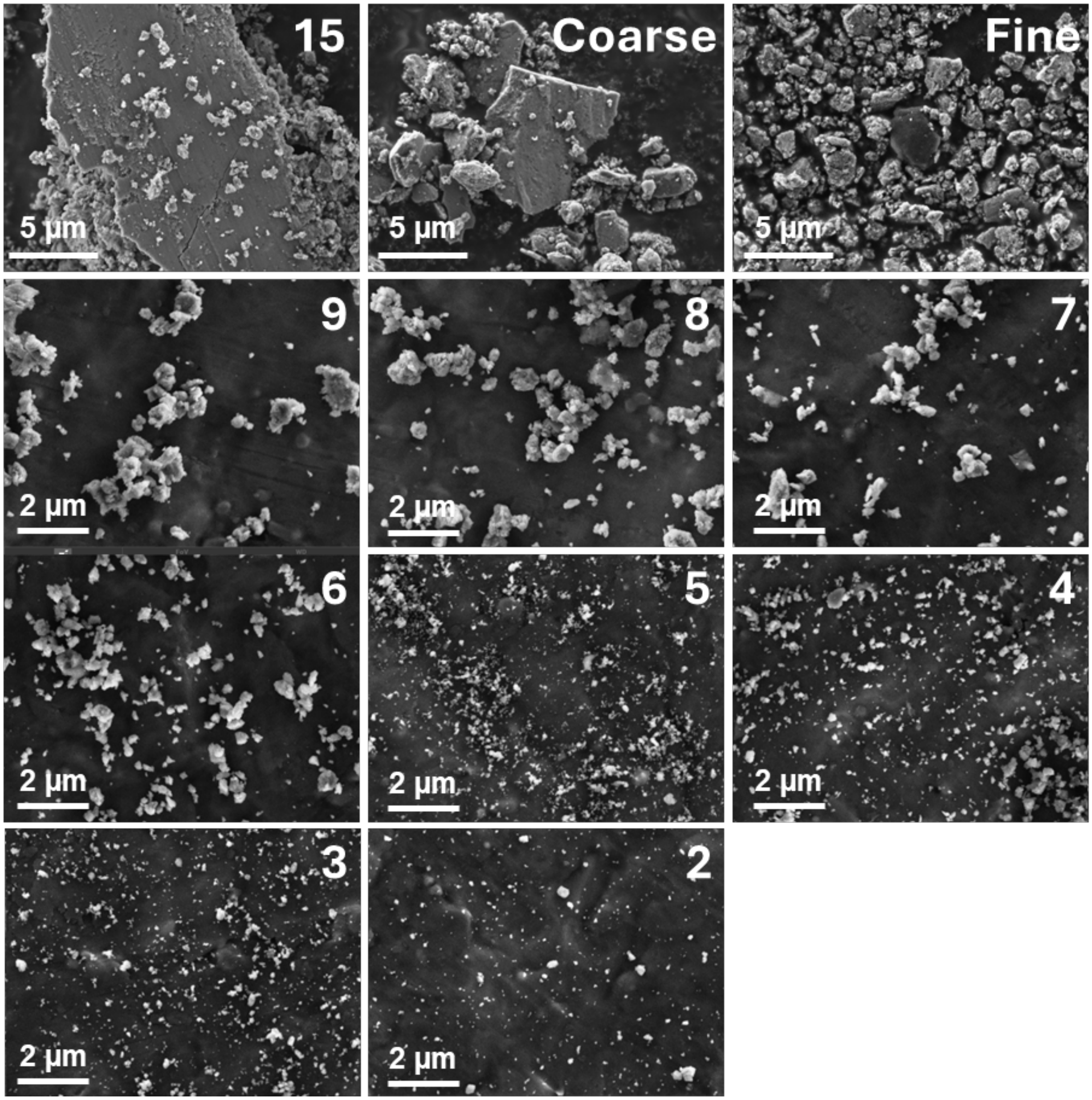

Figure S8.1: SEM images (secondary electrons) taken at 5 keV from brake wear particles generated with LM brake pads. The numbers in the pictures refer to the corresponding stages from the ELPI+. Coarse refers to stages 14, 13, and 12. Fine refers to stages 11 and 10. Stages 2–9 were sampled on ELPI+ aluminum substrates, while fine, coarse, and stage 15 were sampled on carbon tape.

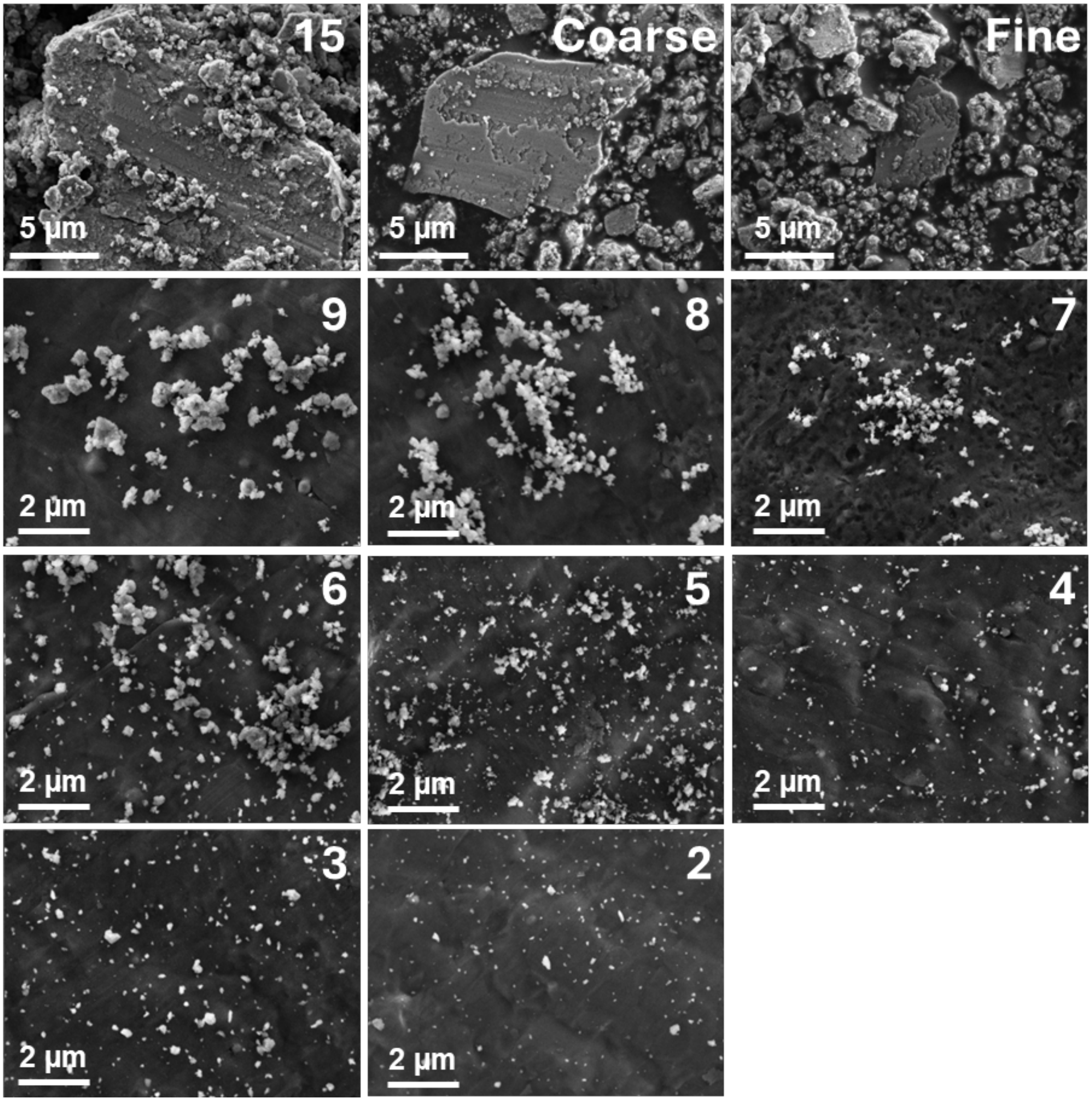

Figure S8.2: SEM images (secondary electrons) taken at 5 keV from brake wear particles generated with NAO brake pads. The numbers in the pictures refer to the corresponding stages from the ELPI+. Coarse refers to stages 14, 13, and 12. Fine refers to stages 11 and 10. Stages 2–9 were sampled on ELPI+ aluminum substrates, while fine, coarse, and stage 15 were sampled on carbon tape.

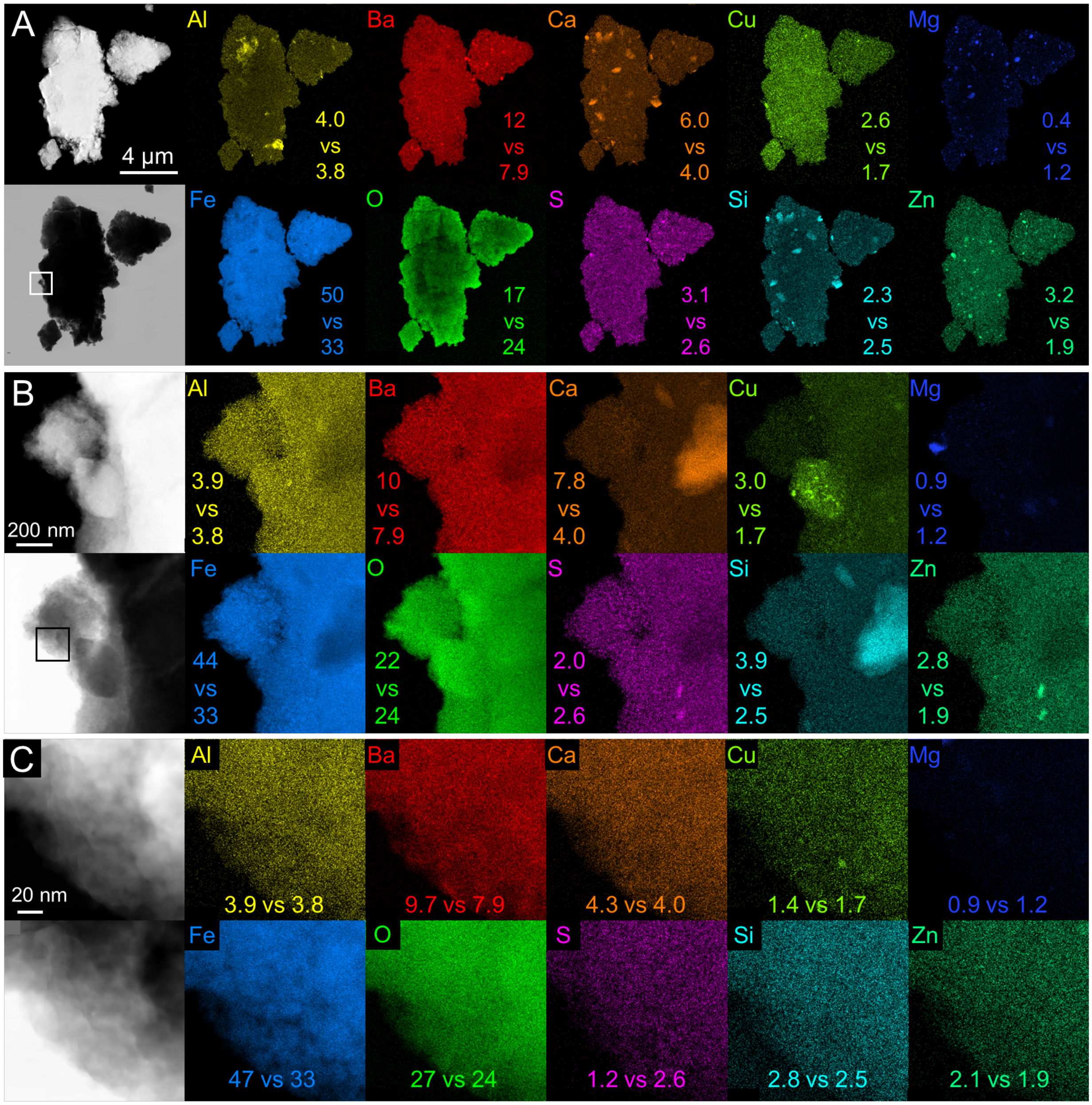

Figure S8.3: (A) HAADF and BF STEM images and their corresponding EDS maps of particles found in the coarse fraction of BWP_{NAO}. The white box in the BF-STEM image from panel (A) is shown with higher magnification in panel (B). Panel (C) refers to a higher magnification of the black box in panel (B). Elemental mass fractions within the field of view according to STEM-EDS are shown in percent versus the bulk according to ICP-OES (or SEM-EDS in case of O, S, and Si).

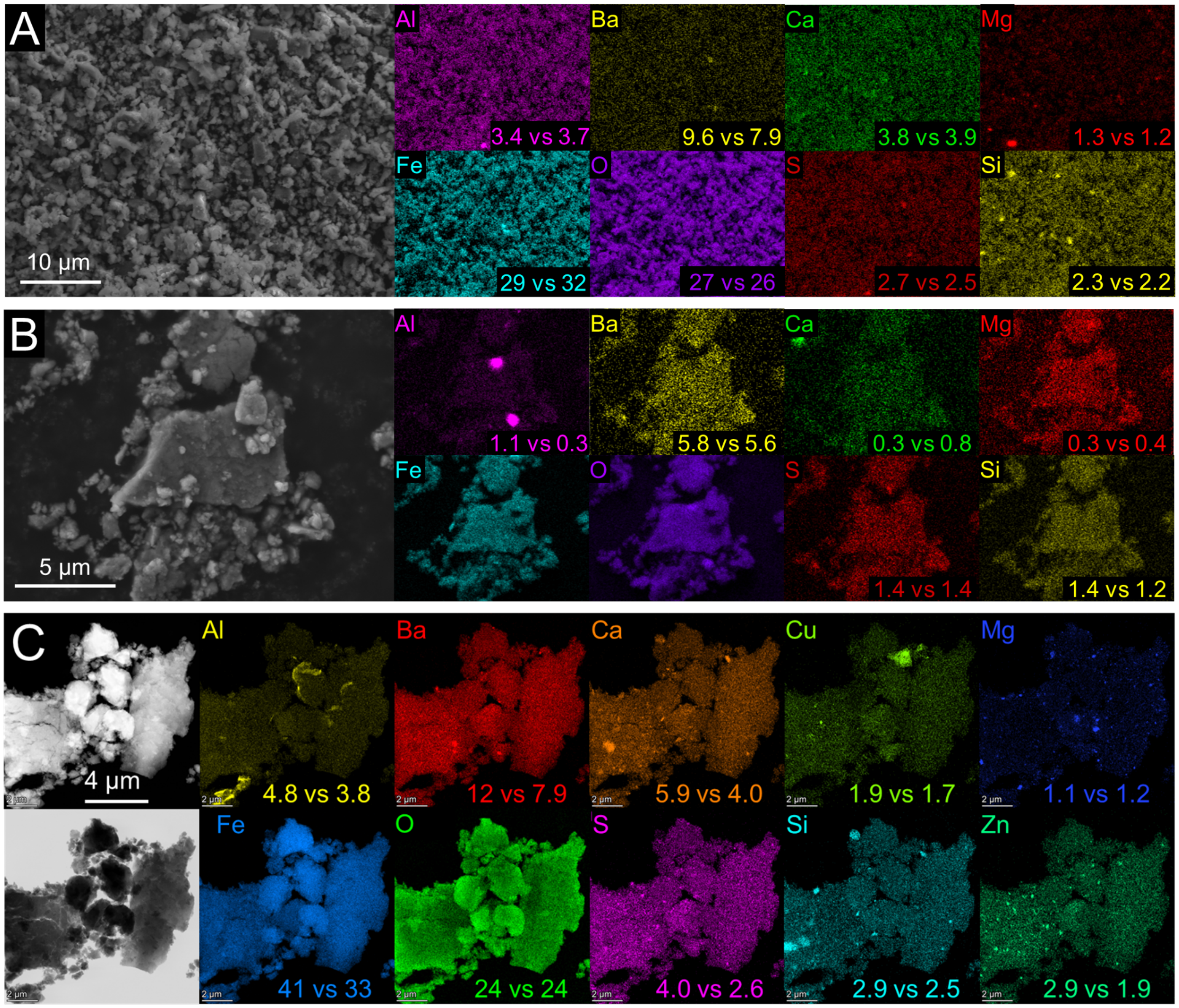

Figure S8.4: SEM images (secondary electrons) with EDS maps of (A) densely packed fine BWP_{NAO} and (B) loosely packed coarse BWP_{LM} on a carbon substrate. Elemental mass fractions within the field of view according to EDS are shown in percent versus the bulk according to ICP-OES (or SEM-EDS in case of O, S, and Si). The EDS data in panel (B) were normalized to an iron mass fraction of 50% due to carbon and oxygen contributions from the background of the loosely packed particles. (C) HAADF and BF STEM images and their corresponding EDS maps of particles found in the coarse fraction of BWP_{NAO}.

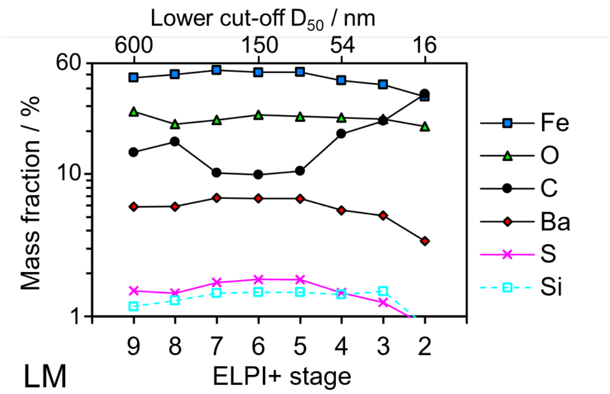

Figure S8.5: Elemental composition of BWP_{LM} in a particle size-separated manner measured with SEM-EDS of BWP_{LM}. The specified particle sizes decrease from stage 9 (600–940 nm) to stage 2 (16–30 nm). Aluminum was excluded from the mass balance because of the aluminum substrates.

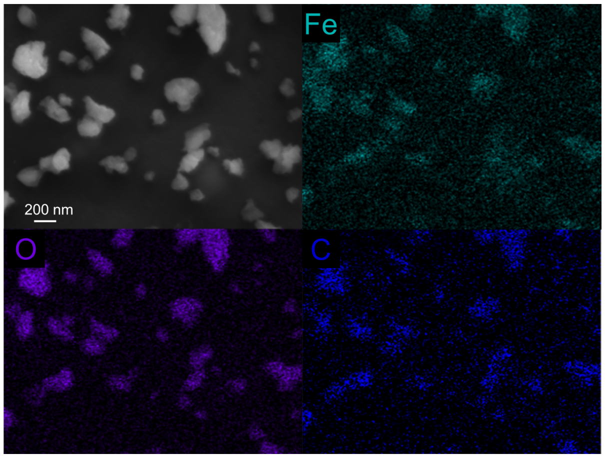

Figure S8.6: SEM image (secondary electrons) with EDS maps of BWP_{NAO} from ELPI+ stage #2.

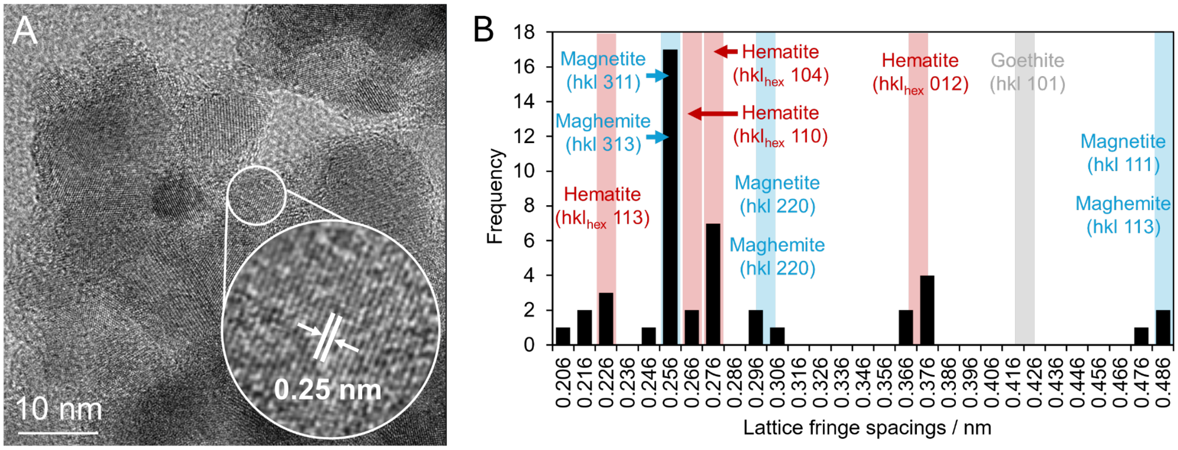

Figure S8.7: (A) High-resolution TEM image from coarse BWP_{LM} with magnified insert showing the lattice fringes. (B) Histogram of lattice fringe spacings measured from high-resolution TEM of 45 different crystals in coarse BWP_{LM}. For comparison, typical bands for magnetite, maghemite, hematite, and goethite are shown according to Cornell and Schwertmann.[18] The labels on the x-axis refer to the upper end of each bin.

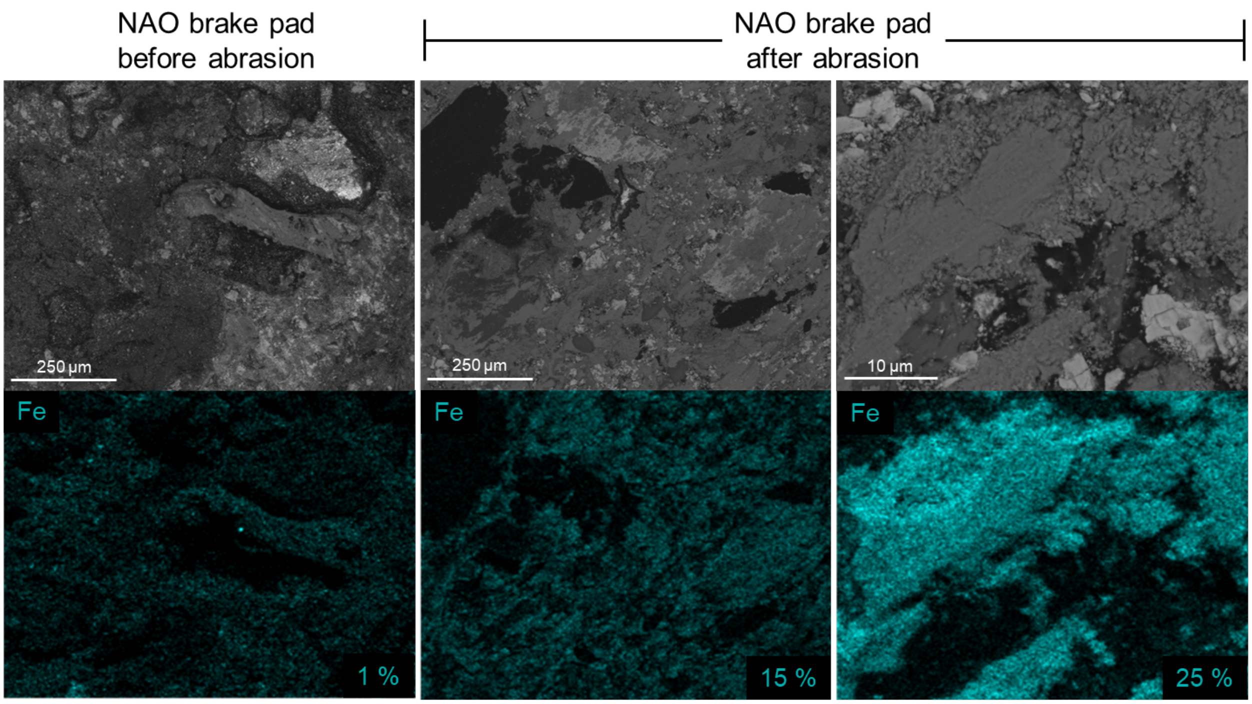

Figure S8.8: SEM images (backscattered electrons) with EDS maps of the NAO brake pad before (left) and after abrasion (center and right). The iron mass fractions according to SEM-EDS are shown in the bottom right corner for each image. The increased level of iron on the NAO brake pad after abrasion is believed to be the result of mass transfer from the disc.

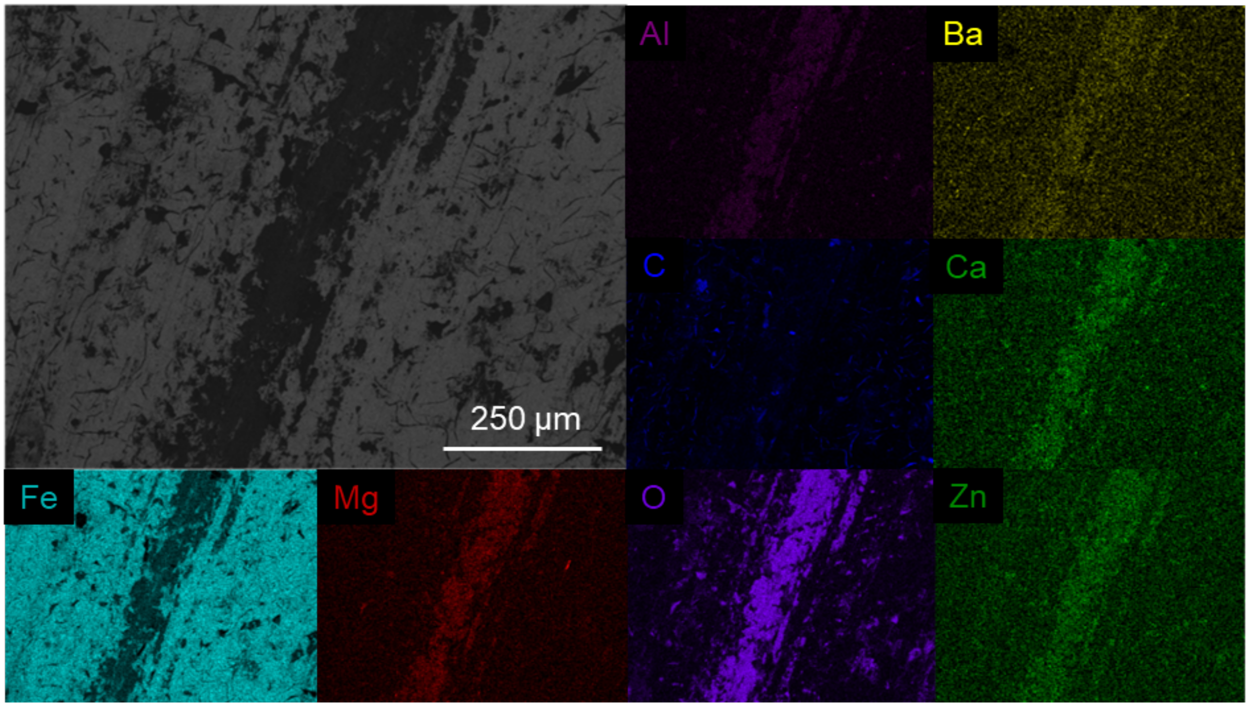

Figure S8.9: SEM image (backscattered electrons) with EDS maps of the brake disc after abrasion with an NAO brake pad. Elements attributable to the brake pad can be found on the brake disc.

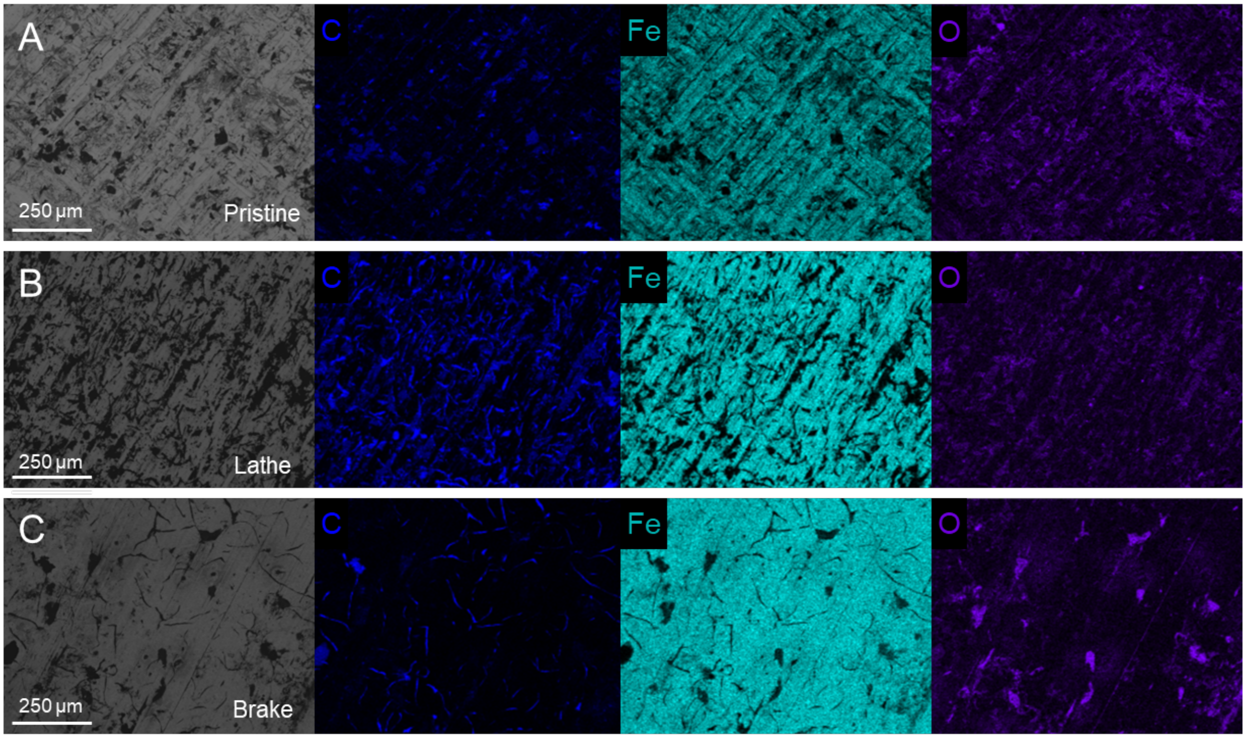

Figure S8.10: SEM images (backscattered electrons) with EDS maps from the brake disc showing the presence of carbon-rich microstructures. The morphology of the carbon differs among different brake disc samples. The (A) pristine brake disc surface shows flake-like carbon. (B) The brake disc surface processed with a lathe is characterized by more elongated carbon structures. (C) Abrasion with a brake pad (in this case NAO) resulted in flake-like and worm-like carbon structures on the brake disc surface. The carbon may undergo physicochemical changes during abrasion.

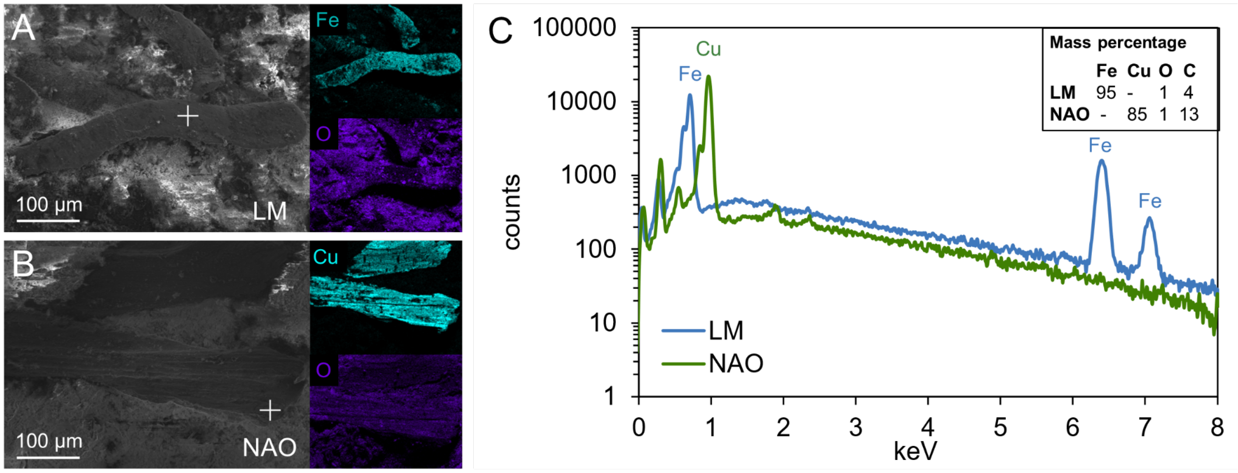

Figure S8.11: SEM images (secondary electrons) with EDS maps from typical fibers in pristine (A) LM and (B) NAO brake pads. The EDS spectra of the points marked with white crosses are shown in panel (C).

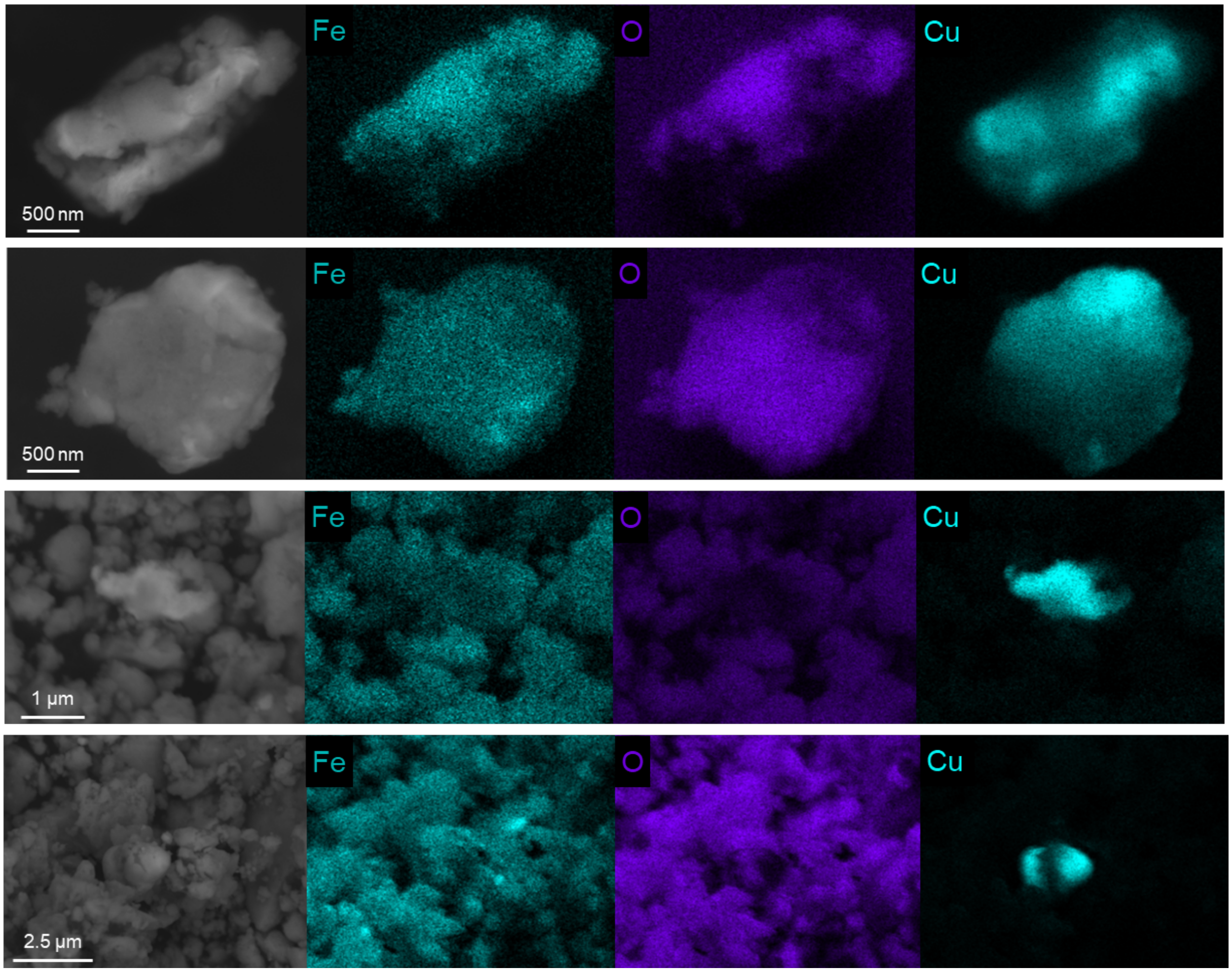

Figure S8.12: SEM images (backscattered electrons) with EDS maps from particles with copper-rich regions found in brake wear particles with NAO but not with LM. In contrast to iron, copper in these particles is interpreted to negatively correlate with oxygen.

Section S9: Iron evaporation and nucleation

Elemental iron (≥ 99.5%, particle size 10 µm, EMSURE®, Supelco) was heated in a tube furnace (Silver Particle Generator, Catalytic Instruments) under a constant HEPA-filtered air flow of 2 L/min. Number concentration and particle size of the outflowing particles were monitored 50 cm downstream of the furnace with an electrostatic classifier (Model 3082, TSI) equipped with a nano-DMA (Model 3085, TSI) and coupled to a condensation particle counter (Model 3752, TSI; d_{50} = 4 nm). Concentrations reported in Figure S9.1 represent the average over a 60-second measurement period once the temperature had stabilized. Size distributions are the average of three scans per temperature setpoint. Error bars in Figure S9.1A indicate the standard deviation of the triplicate measurements.

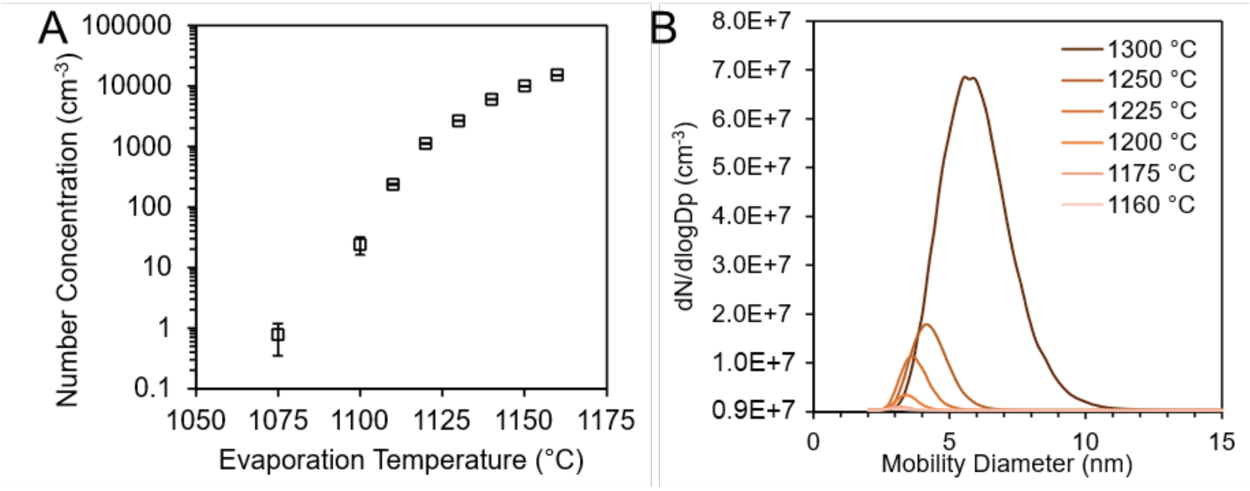

Figure S9.1: (A) Measured number concentrations of polydisperse/non-size-selected airborne nanoparticles evaporated and nucleated from elemental iron as a function of temperature. (B) Corresponding measured particle size distributions.

Section S10: Acellular antioxidant depletion

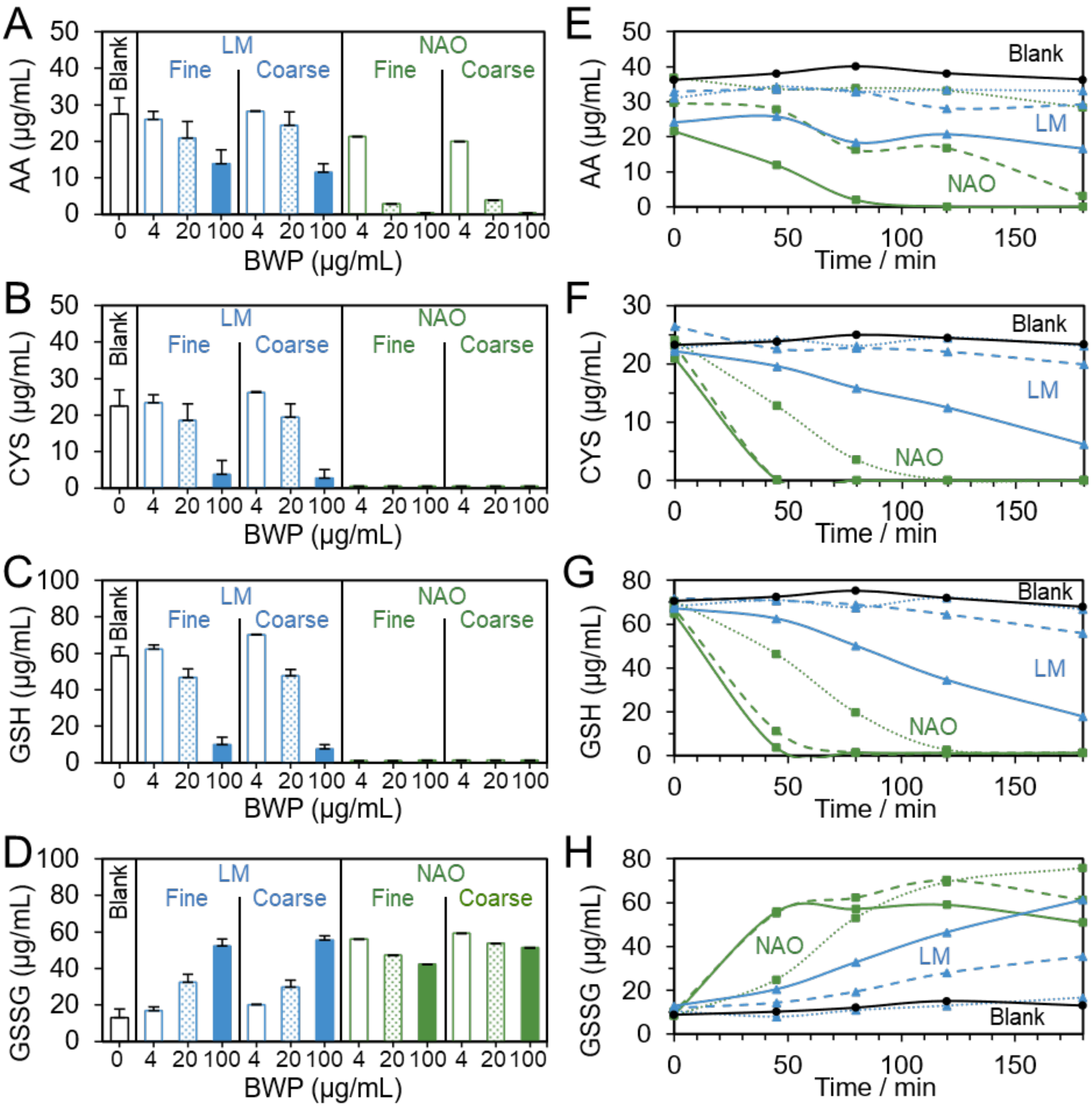

Figure S10.1: Concentrations of acellular (A) ascorbic acid (AA), (B) cysteine (CYS), (C) glutathione (GSH), and (D) glutathione disulfide (GSSG) after 3 h exposure to 4, 20, and 100 µg/mL of BWP_{LM} or BWP_{NAO} in surrogate epithelial lung fluid (SELF) at 37 °C and pH 7.4. The kinetic profiles are shown in panels (E), (F), (G), and (H) for fine BWP_{LM} (triangles) and BWP_{NAO} (squares). The dotted, dashed, and solid lines refer to 4, 20, and 100 µg/mL of BWP, respectively. The solid lines with circles refer to SELF without any BWP.

Section S11: Cytotoxicity

Figure S11.1 shows the effect of exposure on cellular metabolic activity (WST-1 assay). The exposure to the highest concentration of BWP_{LM} was associated with a 31.2% (95% CI: 0.03%, 60.2%) reduction of the metabolic activity, whilst the highest concentrations of BWP_{NAO} fine and coarse were associated with a 31.8% (95% CI: 3.3%, 60.3%) and 34.6% (95% CI: 8.9%, 60.2%) reduction, respectively.

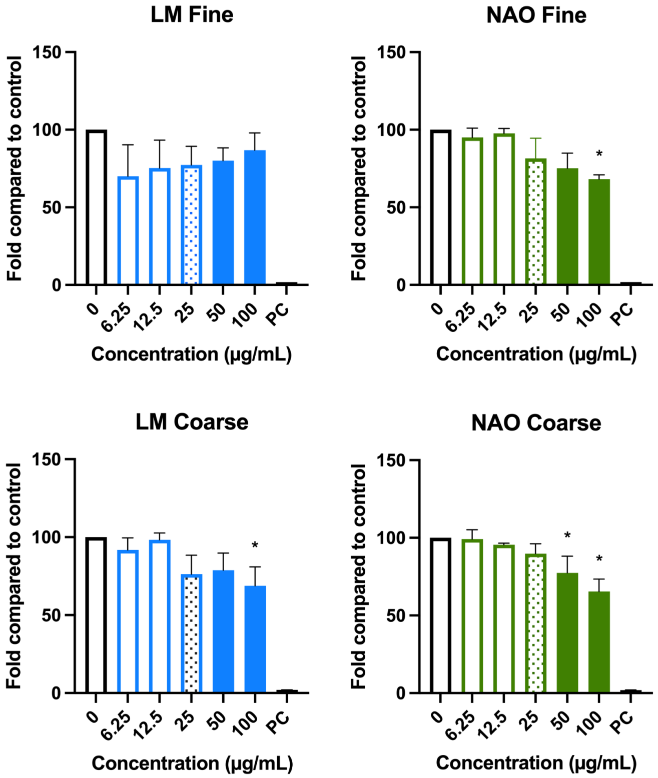

Figure S11.1: Effect of brake wear particle samples on cellular metabolic activity (i.e. WST-1 assay) at 24 h exposure in A549 cells. Bars and error bars are mean and SE of at least three independent experiments. *P < 0.05 compared to control group. PC = Positive Control

Figure S11.2 shows the effect of exposure on cell membrane permeability (LDH activity in cell culture medium). The exposure to the highest concentration of BWP_{NAO} fine and coarse were associated with a 27.1% (95% CI: 4.8%, 49.5%) and 23.2% (95% CI: 2.8%, 43.5%) increased LDH activity in cell culture medium of cells exposed to BWP, respectively. The apparent increase in LDH release observed for BWP_{LM} fine at 6.25–12.5 µg/mL was not statistically significant and likely reflects inter-experimental variability. This observation was not further investigated as the cytotoxicity assays were primarily used to confirm sublethal exposure conditions for the comet assay.

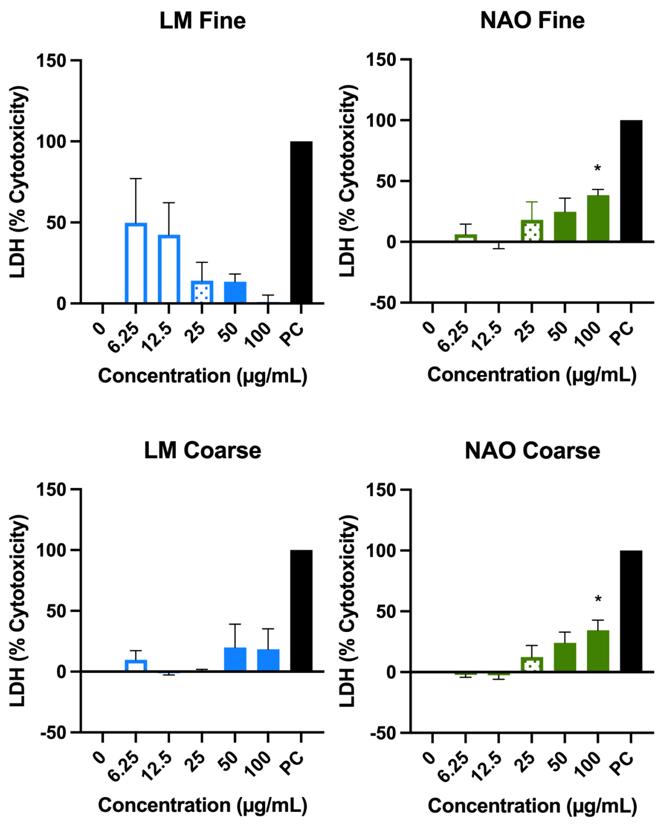

Figure S11.2: Effect of brake wear particles on cell membrane permeability (i.e. lactate dehydrogenase activity in cell culture medium) at 24 h exposure in A549 cells. Bars and error bars are mean and SE of at least three independent experiments. *P < 0.05 compared to control group. PC = Positive Control.

Bibliography

(1) Shahpoury, P.; Harner, T.; Lammel, G.; Lelieveld, S.; Tong, H.; Wilson, J. Development of an Antioxidant Assay to Study Oxidative Potential of Airborne Particulate Matter. *Atmos. Meas. Tech.* **2019**, *12*, 6529–6539. https://doi.org/10.1016/j.atmosenv.2021.118894.

(2) Jensen, K. A.; Kembouche, Y.; Christiansen, E.; Jacobsen, N. R.; Wallin, H.; Guiot, C.; Spalla, O.; Witschger, O. *Deliverable 3: Final Protocol for Producing Suitable MN Exposure Media*; 2011. https://www.anses.fr/en/system/files/nanogenotox_deliverable_6.pdf.

(3) Wils, R. S.; Jacobsen, N. R.; Vogel, U.; Roursgaard, M.; Jensen, A.; Møller, P. Pleural Inflammatory Response, Mesothelin Content and DNA Damage in Mice at One-Year after Intra-Pleural Carbon Nanotube Administration. *Toxicology* **2023**, *499*, 153662. https://doi.org/10.1016/j.tox.2023.153662.

(4) Møller, P.; Azqueta, A.; Sanz-serrano, J.; Bakuradze, T.; Richling, E.; Bankoglu, E. E.; Stopper, H.; Bastos, V. C.; Langie, S. A. S.; Jensen, A.; Scavone, F.; Giovannelli, L.; Wojewódzka, M.; Kruszewski, M.; Valdiglesias, V.; Laffon, B.; Costa, C.; Costa, S.; Teixeira, J. P.; Marino, M.; Del Bo, C.; Riso, P.; Zheng, C.; Shaposhnikov, S.; Collins, A. Visual Comet Scoring Revisited: A Guide to Scoring Comet Assay Slides and Obtaining Reliable Results. *Mutagenesis* **2023**, *38* (5), 253–263. https://doi.org/10.1093/mutage/gead015.

(5) Møller, P. Measurement of Oxidatively Damaged DNA in Mammalian Cells Using the Comet Assay: Reflections on Validity, Reliability and Variability. *Mutat. Res. - Genet. Toxicol. Environ. Mutagen.* **2022**, *873*, 503423. https://doi.org/10.1016/j.mrgentox.2021.503423.

(6) Azqueta, A.; Stopper, H.; Zegura, B.; Dusinska, M.; Møller, P. Cytotoxicity and Cell Death Cause False Positive Results in the in Vitro Comet Assay ? *Mutat. Res. - Genet. Toxicol. Environ. Mutagen.* **2022**, *881*, 503520. https://doi.org/10.1016/j.mrgentox.2022.503520.

(7) Møller, P.; Muruzabal, D.; Bakuradze, T.; Richling, E.; Bankoglu, E. E.; Stopper, H.; Langie, S. A. S.; Azqueta, A.; Jensen, A.; Scavone, F.; Giovannelli, L.; Wojewódzka, M.; Kruszewski, M.; Valdiglesias, V.; Laffon, B.; Costa, C.; Costa, S.; Teixeira, J. P.; Marino, M.; Del Bo, C.; Riso, P.; Shaposhnikov, S.; Collins, A. Potassium Bromate as Positive Assay Control for the Fpg-Modified Comet Assay. *Mutagenesis* **2020**, *35* (4), 341–348. https://doi.org/10.1093/mutage/geaa011.

(8) Møller, P.; Azqueta, A.; Rodriguez-Garraus, A.; Bakuradze, T.; Richling, E.; Bankoglu, E. E.; Stopper, H.; Bastos, V. C.; Langie, S. A. S.; Jensen, A.; Ristori, S.; Scavone, F.; Giovannelli, L.; Wojewódzka, M.; Kruszewski, M.; Valdiglesias, V.; Laffon, B.; Costa, C.; Costa, S.; Teixeira, J. P.; Marino, M.; Del Bo, C.; Riso, P.; Zheng, C.; Shaposhnikov, S.; Collins, A. Long-Term Cryopreservation of Potassium Bromate Positive Assay Controls for Measurement of Oxidatively Damaged DNA by the Fpg-Modified Comet Assay: Results from the HCOMET Ring Trial. *Mutagenesis* **2023**, *38* (5), 264–272. https://doi.org/10.1093/mutage/gead020.

(9) Wörle-Knirsch, J. M.; Pulskamp, K.; Krug, H. F. Oops They Did It Again ! Carbon Nanotubes Hoax Scientists in Viability Assays. *Nano Lett.* **2006**, *6* (6), 1261–1268. https://doi.org/10.1021/nl060177c.

(10) Danielsen, P. H.; Cao, Y.; Roursgaard, M.; Møller, P.; Loft, S. Endothelial Cell Activation, Oxidative Stress and Inflammation Induced by a Panel of Metal-Based Nanomaterials. *Nanotoxicology* **2015**, *9* (7), 813–824. https://doi.org/10.3109/17435390.2014.980449.

(11) Wils, R. S.; Jacobsen, N. R.; Di Ianni, E.; Roursgaard, M.; Møller, P. Reactive Oxygen Species Production , Genotoxicity and Telomere Length in FE1-Muta ™ Mouse Lung Epithelial Cells Exposed to Carbon Nanotubes. *Nanotoxicology* **2021**, *15* (5), 661–672. https://doi.org/10.1080/17435390.2021.1910359.

(12) Vesterdal, L. K.; Mikkelsen, L.; Folkmann, J. K.; Sheykhzade, M.; Cao, Y.; Roursgaard, M.; Loft, S.; Møller, P. Carbon Black Nanoparticles and Vascular Dysfunction in Cultured Endothelial Cells and Artery Segments. *Toxicol. Lett.* **2012**, *214* (1), 19–26. https://doi.org/10.1016/j.toxlet.2012.07.022.

(13) Di Ianni, E.; Jacobsen, N. R.; Vogel, U. B.; Møller, P. Systematic Review on Primary and Secondary Genotoxicity of Carbon Black Nanoparticles in Mammalian Cells and Animals. *Mutat. Res. Mutat. Res.* **2022**, *790*, 108441. https://doi.org/10.1016/j.mrrev.2022.108441.

(14) Liu, Y.; Møller, P.; Roursgaard, M. Aminated Polystyrene and DNA Strand Breaks in A549, Caco-2, THP-1 and U937 Human Cell Lines. *Mutat. Res. - Genet. Toxicol. Environ. Mutagen.* **2025**, *903*, 503865. https://doi.org/10.1016/j.mrgentox.2025.503865.

(15) Di Ianni, E.; Møller, P.; Vogel, U. B.; Jacobsen, N. R. Pro-Inflammatory Response and Genotoxicity Caused by Clay and Graphene Nanomaterials in A549 and THP-1 Cells. *Mutat. Res. - Genet. Toxicol. Environ. Mutagen.* **2021**, *872*, 503405. https://doi.org/10.1016/j.mrgentox.2021.503405.

(16) Di Ianni, E.; Erdem, J. S.; Møller, P.; Sahlgren, N. M.; Poulsen, S. S.; Knudsen, K. B.; Zienolddiny, S.; Saber, A. T.; Wallin, H.; Vogel, U.; Jacobsen, N. R. In Vitro-in Vivo Correlations of Pulmonary Inflammogenicity and Genotoxicity of MWCNT. *Part. Fibre Toxicol.* **2021**, *18* (25). https://doi.org/10.1186/s12989-021-00413-2.

(17) Di Ianni, E.; Møller, P.; Cholakova, T.; Wolff, H.; Jacobsen, N. R.; Vogel, U. Assessment of Primary and Inflammation-Driven Genotoxicity of Carbon Black Nanoparticles in Vitro and in Vivo. *Nanotoxicology* **2022**, *16* (4), 526–545. https://doi.org/10.1080/17435390.2022.2106906.

(18) Cornell, R. M.; Schwertmann, U. *The Iron Oxides: Structure, Properties, Reactions, Occurences and Uses*, 2nd ed.; WILEY-VCH Verlag GmbH & Co. KGaA: Weinheim, 2003.
